# Origin of Secretin Receptor Precedes the Advent of Tetrapoda: Evidence on the Separated Origins of Secretin and Orexin

**DOI:** 10.1371/journal.pone.0019384

**Published:** 2011-04-29

**Authors:** Janice K. V. Tam, Kwan-Wa Lau, Leo T. O. Lee, Jessica Y. S. Chu, Kwong-Man Ng, Alain Fournier, Hubert Vaudry, Billy K. C. Chow

**Affiliations:** 1 School of Biological Sciences, Research Centre of Heart, Brain, Hormone and Healthy Ageing, Li Ka Shing Faculty of Medicine, The University of Hong Kong, Pokfulam, Hong Kong SAR, China; 2 Stem Cell & Regenerative Medicine Program, Research Centre of Heart, Brain, Hormone and Healthy Ageing, Li Ka Shing Faculty of Medicine, The University of Hong Kong, Pokfulam, Hong Kong SAR, China; 3 INRS – Institut Armand-Frappier, Université du Quebec, Laval, Québec, Canada; 4 INSERM U982, European Institute for Peptide Research, University of Rouen, Mont-Saint-Aignan, France; University of Wyoming, United States of America

## Abstract

At present, secretin and its receptor have only been identified in mammals, and the origin of this ligand-receptor pair in early vertebrates is unclear. In addition, the elusive similarities of secretin and orexin in terms of both structures and functions suggest a common ancestral origin early in the vertebrate lineage. In this article, with the cloning and functional characterization of secretin receptors from lungfish and *X. laevis* as well as frog (*X. laevis* and *Rana rugulosa*) secretins, we provide evidence that the secretin ligand-receptor pair has already diverged and become highly specific by the emergence of tetrapods. The secretin receptor-like sequence cloned from lungfish indicates that the secretin receptor was descended from a VPAC-like receptor prior the advent of sarcopterygians. To clarify the controversial relationship of secretin and orexin, orexin type-2 receptor was cloned from *X. laevis*. We demonstrated that, in frog, secretin and orexin could activate their mutual receptors, indicating their coordinated complementary role in mediating physiological processes in non-mammalian vertebrates. However, among the peptides in the secretin/glucagon superfamily, secretin was found to be the only peptide that could activate the orexin receptor. We therefore hypothesize that secretin and orexin are of different ancestral origins early in the vertebrate lineage.

## Introduction

Based on structural similarity, secretin (SCT) is classified to the secretin/glucagon superfamily that also includes vasoactive intestinal peptide (VIP), pituitary adenylate cyclase-activating polypeptide (PACAP), PACAP-related peptide (PRP), glucagon, peptide-histidine-isoleucine (PHI), glucagon-like peptides (GLP-1 and GLP-2), gastric inhibitory polypeptide (GIP), and growth hormone-releasing hormone (GHRH) [1]. It has been suggested that several gene and exon duplication events followed by subsequent modifications of an ancestral gene have given rise to these structurally similar peptides [2], [3]. Secretin was first discovered as a gastrointestinal hormone with its function in stimulating pancreatic flow [4]. Recently, various roles of secretin in the central and peripheral nervous system as well as in other organs including pituitary, kidney, intestine and heart [5]–[9] have further been proposed.

Secretin carries out its hormonal actions through the secretin receptor (SCTR), which is a member of Class II B1 guanine nucleotide binding protein (G protein)-coupled receptors (GPCR) [6]. This class of GPCR utilizes intracellular second messengers including cyclic AMP and calcium ions in signaling pathways (For details, see review [9]). The first secretin receptor was isolated from a rat NG108-15 cell line based on its high affinity for secretin in transfected COS cells [5]. Subsequently, secretin receptors were cloned from several mammalian species, including human [10]–[12], mouse [13], rat [14], bovine [15] and rabbit [16].

Orexins A and B [17] or hypocretins 1 and 2 [18] are peptides isolated from the rat hypothalamus in 1998 by two independent research groups. Both peptides are derived from the same precursor protein and are produced by differential proteolytic cleavage. Because the C-terminal portions of both orexin peptides resemble the N-terminal of secretin, orexins were proposed to have originated from secretin or the related peptides in the secretin/glucagon superfamily [18], [19]. This hypothesis was then examined by studying the bindings of these peptides with their receptors in mammals and conflicting results were reported. Porcine SCT was found to displace the binding of [125^I^] orexin A in the rat anterior hypothalamus and orexin receptor-transfected cells [20]. On the contrary, another research group showed that SCT was unable to displace [125^I^] orexin A or induce calcium elevation in human orexin type-2 receptor-transfected CHO cells [21]. There were also reports indicating that SCT exhibited neither agonistic nor antagonistic effects on the human orexin receptors [22]. To date, orexins have been identified in several jawed vertebrates, including teleosts (pufferfish and zebrafish) [23], frog [24], chicken [25] and mammals [18]. Two orexin receptors encoded by separate genes were found in mammals [17], but in zebrafish [23] and chicken [26], only type-2 receptors were isolated. Functionally, orexins are neuropeptides that modulate energy homeostasis, feeding behavior [27]–[29], gastrointestinal secretion [30]–[32], sleep-wake cycle [33], and drinking behavior [34]; and it is interesting to note that some of the effects of orexin overlap with those of secretin [35].

To our knowledge, secretin and secretin receptors have only been functionally identified in mammals while a secretin-like peptide sequence has been isolated in chicken [36]–[38]. To understand the evolutionary history of secretin and secretin receptor, we have chosen the African lungfish *Protopterus dolloi* and two frog species (*Xenopus laevis* and *Rana rugulosa*) for the isolation of SCT and SCTR homologues as they are extant species in the Sarcopterygii lineage [39]. Lungfish and the fish ancestors of the tetrapod lineage are believed to be originated within a short time window of about 20 million years, back in the early Devonian (about 380 to 400 million years ago) [40]. Hence, lungfish holds an important evolutionary position in the vertebrate lineage extending from the Paleozoic fishes to the tetrapods [38]. Frog species diversified and radiated in the amphibian lineage, marking the critical point of Devonian origin of tetrapods from the transition of aquatic to terrestrial habitats [41], [42]. In the present study, we have cloned and functionally characterized putative SCTRs from lungfish and frogs, showing for the first time that a SCTR-like sequence was already present in the lobe-finned fish dating back to the early Devonian. Functional studies evidently showed that these putative SCTRs were coupled to downstream signaling mechanisms involving intracellular cAMP and calcium ions.

Because of the elusive structural and functional similarities observed in secretin and orexin peptides in mammals, together with the conflicting reports on the cross-reactivity of secretin and orexin with their mutual receptors, we sought to test the ligand-receptor activation of secretin and orexin in *X. laevis* that now remains confined to mammalian studies. We hypothesized that secretin and orexin receptors could have been functional complementary partners in mediating physiological processes before the origin of mammals; and subsequent to the early divergence of mammals, they became highly specific to their respective ligands. Our expectation under this hypothesis is that secretin and orexin could activate their mutual receptors in frog species, but not in mammalians. Therefore, in addition to secretin and secretin receptor, the orexin type-2 receptor was also cloned from *X. laevis* to clarify the ancestral relationship of secretin and orexin. We showed that *Xenopus* orexin A (xOA) could stimulate calcium transients in both lungfish and *X. laevis* SCTRs; while *Xenopus* secretin (xSCT) could also evoke calcium elevations in *Xenopus* orexin type-2 receptor (xOX2R). Substantiated by these reciprocal ligand-receptor activations in non-mammalian vertebrates, we provide evidence that, secretin and orexin, could be modulating physiological processes in coordination before the divergence of mammals; but we found that such interaction was due to their moderate structural identities instead of a common ancestral origin early in the vertebrate lineage.

## Results

### Identification and Analysis of Putative Secretin Receptors in *P. dolloi* and *X. laevis*

**Figure 1:**
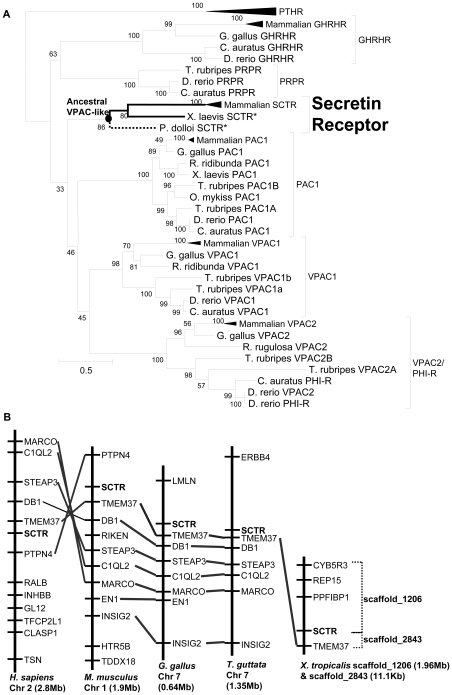
Analyses of secretin receptor phylogeny and *in silico* genomic locations. (A) Receptor phylogeny: phylogenetic analysis of vertebrate receptors in Class II B1 GPCR. The tree was generated by Maximum Likelihood (ML) and plotted by MEGA 5.0. Receptors cloned in the present study are marked by an asterisk. Diverged from the ancestral VPAC-like receptor (denoted by a black dot), the *P. dolloi* SCTR retained the VIP/PACAP functions (branch in dotted line); whereas the *X. laevis* and mammalian SCTRs acquired the specificity towards secretin (branch in thick solid black line). PTHR, parathyroid hormone receptor; SCTR, secretin receptor; PAC1, pituitary adenylate cyclase-activating polypeptide (PACAP) receptor type I; VPAC1, vasoactive intestinal peptide (VIP)-PACAP receptor I; VPAC2, VIP-PACAP receptor II. (B) Chromosomal locations of secretin receptor in various vertebrate species. Genes adjacent to secretin receptor in different vertebrate genomes are shown. Homologous genes present in different species are linked to show their similarities in chromosomal location.

By searching the *X. tropicalis* genome, DNA sequences that shared high levels of sequence identity with mammalian SCTRs were identified. Primers were designed accordingly to amplify the putative SCTR in *X. laevis*, and a full-length cDNA of 1841 bp (GenBank accession no. HQ236552) with an open reading frame of 1350 bp encoding a 450-amino acid protein was obtained (Fig. S1). To amplify the SCTR in *P. dolloi*, degenerative primers were designed. The full-length putative *P. dolloi* SCTR cDNA was 1509 bp (GenBank accession no. HQ236551) with an open reading frame of 1389 bp encoding a 463-amino acid protein (Fig. S2). Using the CBS Prediction Servers (http://www.cbs.dtu.dk/services/), putative lfSCTR and xSCTR were shown to structurally resemble other class II B1 GPCRs with an N-terminal signal peptide of 28 and 24 amino acids, respectively, a ligand binding domain and seven transmembrane domains. Phylogenetic analyses of putative lfSCTR and xSCTR were performed using the Maximum Likelihood method with the Jones-Taylor-Thornton (JTT) model (Fig. 1A). PAC1, VPAC1 and VPAC2 receptors were included for their closer phylogenetic relationships with SCTRs [8], while PTHR was used as an outgroup. The tree grouped the putative lfSCTR and xSCTR to the clade of SCTRs, supporting their identities as the orthologs of mammalian SCTRs. Moreover, phylogenies inferred from the SCTR sub-branch are consistent with the divergence of vertebrate groups. The overall tree containing the analyzed receptors is in agreement with the published observations [43]. When compared to human, rat, mouse, rabbit, and bovine SCTR, lfSCTR and xSCTR shared 58.8–71.0% sequence identity in the ligand-binding domain (Fig. S3A), and 62.6–69.0% identity for the entire receptor (Fig. S3B). Structurally, the six cysteine residues involved in the formation of three disulphide linkages within the ectodomain [44], [45] and the third endoloop (IC_3_), as well as the RLAR/K motif for Gs coupling [46] were conserved in lfSCTR and xSCTR (Fig S4). Interestingly, the IIRIL motif that was believed to be unique to VPAC1 receptors [47] was found in all secretin receptors. Moreover, the PDI/V motif present in all VIP receptors [47] was also found in lfSCTR. In chromosomal synteny analysis (Fig. 1B), SCTR genes were located in close proximity to the Tmem37 genes in all the analyzed species. This information therefore supports that the SCTR gene in *X. tropicalis* is homologous to the mammalian SCTR genes.

### Molecular Cloning of *X. laevis* and *R. rugulosa* Secretin Precursors

**Figure 2:**
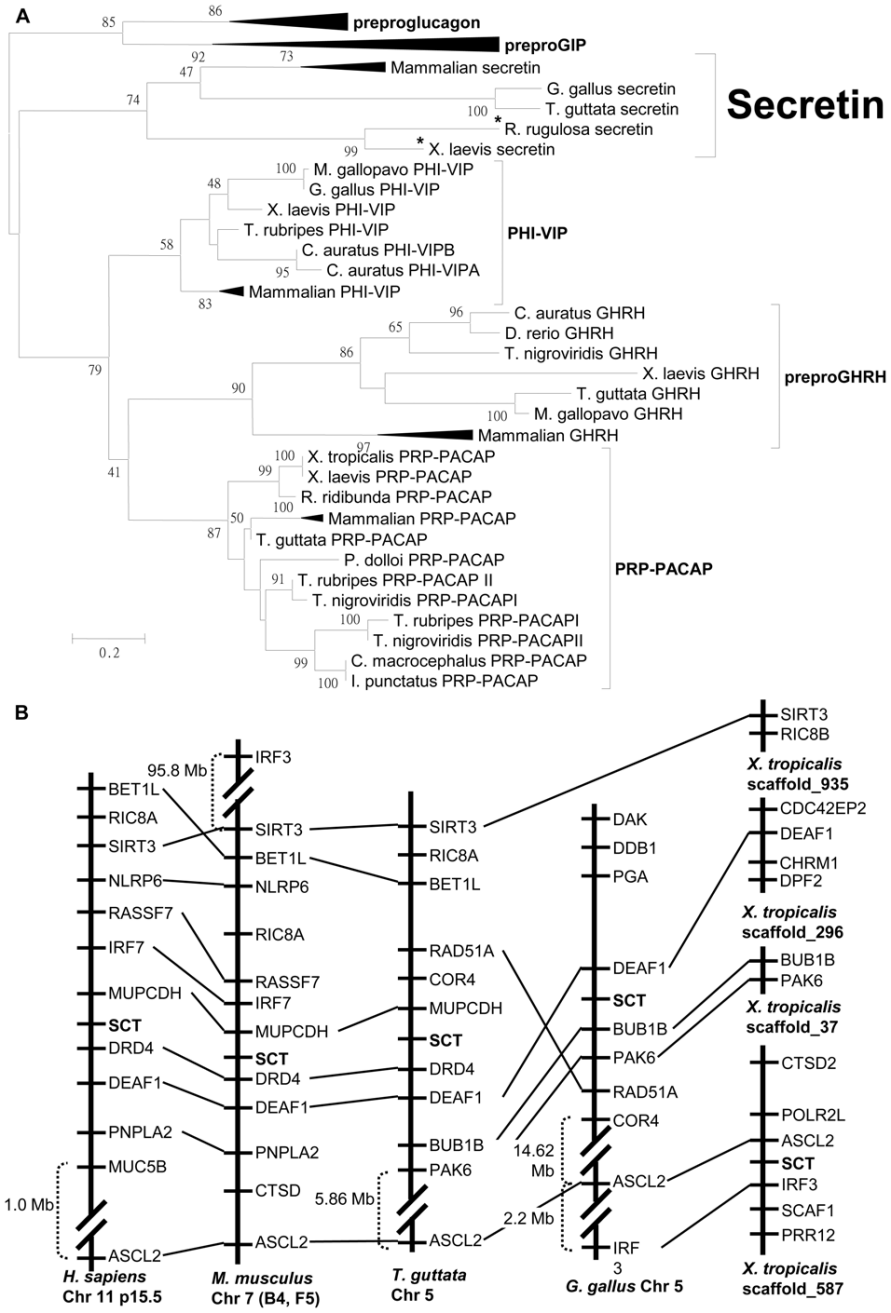
Analyses of secretin phylogeny and *in silico* genomic locations. (A) Ligand Phylogeny: phylogenetic analysis of the secretin/glucagon hormone precursor superfamily. The tree was generated by Maximum Likelihood (ML) and plotted by MEGA 5.0. Sequences determined in the present study are marked by an asterisk. SCT, secretin precursor; preproGHRH, prepro-growth hormone-releasing hormone; PHI-VIP, peptide histidine isoleucine-vasoactive intestinal peptide precursor; PRP-PACAP, pituitary adenylate cyclase-activating polypeptide (PACAP)-related peptide-PACAP precursor. (B) Chromosomal locations of secretin genes in various vertebrate species. Neighboring genes of secretin in different vertebrate genomes are shown. Homologous genes in proximity of secretin are linked by straight lines to demonstrate the syntenic gene environment of secretin in the analyzed vertebrate species.

The predicted amino acid sequences of *Xenopus* and *Rana* SCT precursors are shown in Fig. S5, S6 (*X. laevis* secretin (xSCT) cDNA GenBank accession no. HQ236553 and *Rana* secretin (Rana SCT) cDNA GenBank accession no. HQ236554). Both precursors encode a 28-amino acid secretin peptide predicted by the conserved GKR motif as cleavage site. Alignment of frog SCT with other SCTs showed identical residues at positions 1, 3, 4, 7, 8, 9 11 and 17 (Fig. S7A) and 57.1–96.4% sequence identity (Fig. S7B). Rana SCT and the predicted *X. tropicalis* SCT are identical while they differ from xSCT by 1 residue at position 5. The phylogenetic relationship of SCT precursors was analyzed with the precursors of the other secretin/glucagon superfamily peptides (Fig. 2A). Precursors instead of mature peptides were used in the analysis because the mature peptides are too short for phylogenetic studies (less than 50 amino acid residues). In the tree, frog, avian and mammalian SCT precursors formed three sub-branches in the same clade, which are consistent with the established phylogenies of these species. In an attempt to analyze the SCT genes in vertebrates, their genomic locations in human, mouse, bird, chicken and *X. tropicalis* were mapped (Fig. 2B). The overall genomic arrangement of the SCT loci was syntenic, as shown by genes such as DRD4, DEAF1, and ASCL2, although the gene environment of SCT in *X. tropicalis* was less conserved comparatively. This could be attributed to the incomplete assembling of scaffolds in the *X. tropicalis* genome, or it is reflecting the occurrence of some major evolutionary events that took place in amphibian divergence. Despite our efforts, we could neither identify a secretin-like sequence in *P. dolloi* by molecular cloning, nor any secretin-like sequence in various fish genome databases by informatics.

### Comparison of Functional Properties of lfSCTR and xSCTR

**Figure 3:**
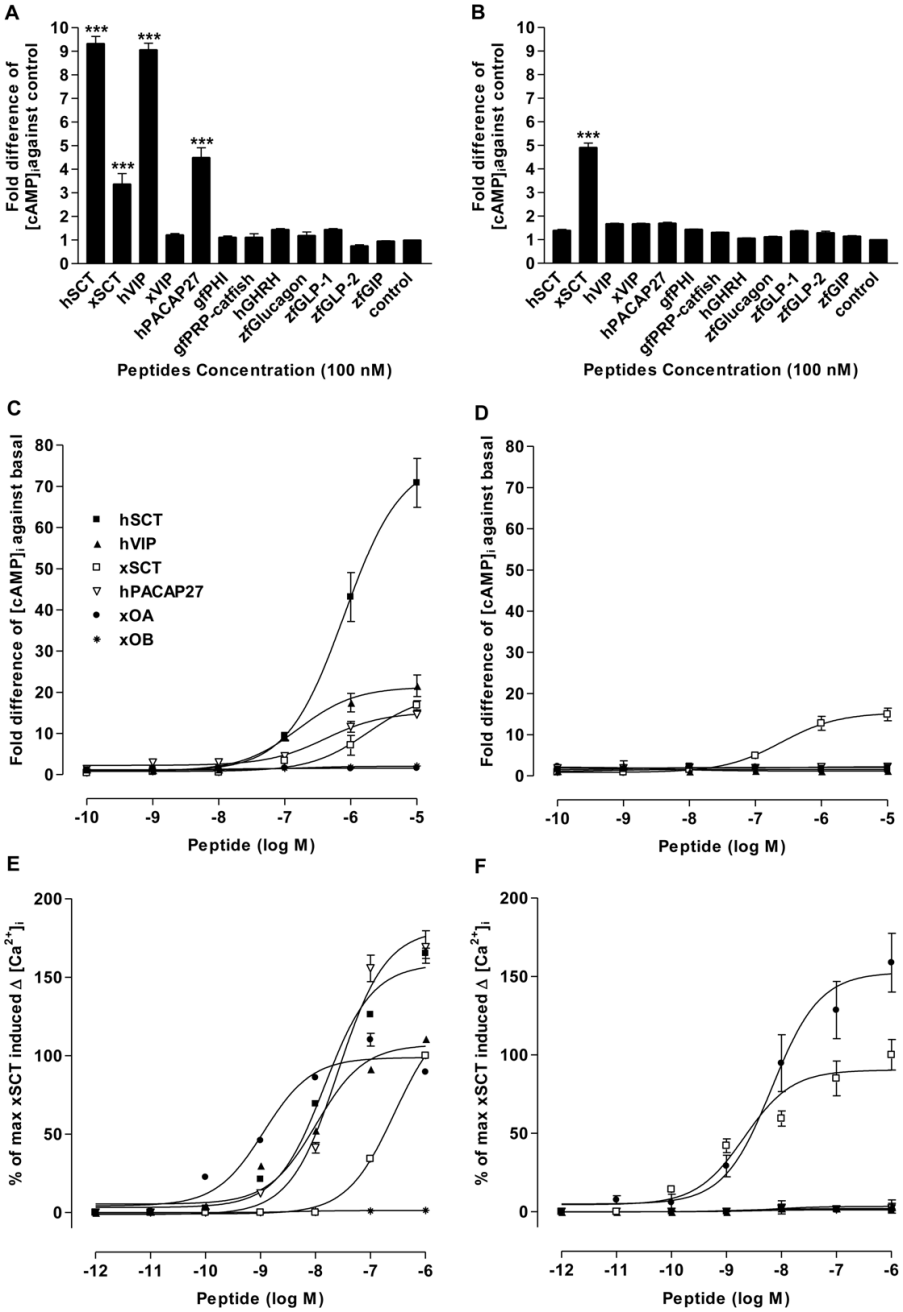
Functional characterization of lfSCTR and xSCTR. Intracellular cAMP accumulation ([cAMP]_i_) in response to 100 nM of the secretin and related peptides on CHO-K1 cells transfected with (A) lfSCTR and (B) xSCTR (*** indicates *P*<0.001). Effects of graded concentrations of peptides on (C) lfSCTR- and (D) xSCTR-expressing cells. Peptide species: h, human; x, *X. laevis*, zf, zebrafish *Danio rerio*; gf, goldfish *Carassius auratus*. Values represent mean ± SEM (n = 4). Effects of secretin and related peptides on intracellular calcium mobilization ([Ca^2+^]_i_) in recombinant CHO cells expressing (E) lfSCTR and (F) xSCTR. Transiently transfected cells expressing the receptors were stimulated with graded concentrations of peptides. Data were expressed in ΔRFU value (maximum changes in the fluorescence signals from baseline) and converted to percentage of the maximum of xSCT-induced [Ca^2+^]_i_ elevation. Results are expressed as mean ± SEM from at least 10 independent experiments, cell number = 20 to 50.

To functionally characterize the putative lfSCTR and xSCTR, CHO cells transiently transfected with these receptors were stimulated by SCT or related peptides, and intracellular cAMP production ([cAMP]_i_) as well as intracellular Ca^2+^ mobilization ([Ca^2+^]_i_) were monitored. Among all the peptides tested, hSCT, xSCT, hVIP, and hPACAP27 (100 nM) were able to significantly stimulate lfSCTR (Fig. 3A). The xSCTR was found highly specific to xSCT in both functional assays (Fig. 3B, 3D and 3F) ([cAMP]_i_ EC_50_ = 0.24 µM and [Ca^2+^]_i_ EC_50_ = 2.52 nM). Graded concentrations of these peptides stimulated both SCTRs dose-dependently (Fig. 3C and D). As shown by the EC_50_ values, the order of specificity in activating lfSCTR was hVIP (0.16 µM)>hPACAP27 (0.41 µM)>hSCT (0.79 µM)>xSCT (1.82 µM). Despite that the peptides were weakly potent as shown by their sub-micromolar EC_50_ values, their abilities to stimulate in a concentration-dependent manner cAMP response showed that they were fully efficacious agonists of this receptors. Of interest is the agonistic effect of hSCT on lfSCTR stimulation. While hSCT was less potent than hVIP and hPACAP27 in activating lfSCTR, at micro-molar concentrations (10^−5^ and 10^−6^ M), hSCT was 3.6 and 5.0 fold more efficacious than hVIP and hPACAP27 respectively in triggering [cAMP]_i_ in lfSCTR-CHO cells. In addition to cAMP stimulation, exposure of lfSCTR-transfected CHO cells preloaded with the Ca^2+^-sensitive dye Fluo-3 to hSCT, xSCT, hVIP, and hACAP27 evoked transient [Ca^2+^]_i_ increase in a dose-dependent manner (Fig. 3E). As shown by their EC_50_ values, the order of specificity was hVIP (10.3 nM)>hSCT (14.7 nM)>hPACAP27 (25.6 nM)>xSCT (273.1 nM), which is in agreement with the cAMP assay. By confocal imaging, real-time traces from single cells were recorded (Fig. S8). Ionomycin, an ionophore, was added at the end of each trial to ensure that [Ca^2+^]_i_ elevation was not caused by cell lysis.

### Tissue Expression of *X. laevis* secretin, *P. dolloi* and *X. laevis* Secretin Receptors

**Figure 4:**
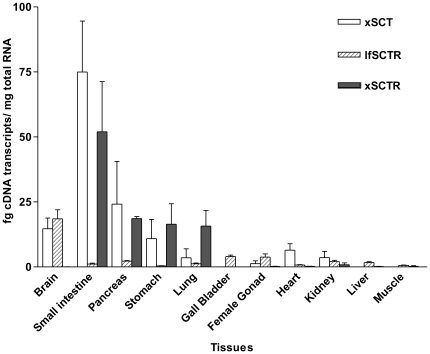
Tissue expression profile of xSCT, lfSCTR and xSCTR. Using real-time RT-PCR, the tissue distribution patterns of lfSCTR, xSCT and xSCTR were investigated on *P. dolloi* and *X. laevis*. The expression level of each gene was calculated from respective standard curve. Data are expressed as mean ± SEM (n = 4).

To examine their potential sites of action, the expression profiles of xSCT, xSCTR and lfSCTR transcripts were studied by the quantitative PCR technique (Fig. 4). All the transcripts were found widely expressed in lungfish and *X. laevis*, and the *X. laevis* intestine exhibited the highest co-expression of SCT and SCTR. This co-expression extended to the gastrointestinal tract (stomach and pancreas), lung and kidney. Expression of the xSCT transcript was also detected in the brain, while its receptor was relatively weakly expressed in this tissue. Unlike the most abundant expression of xSCTR in the digestive system, lfSCTR had the highest transcript level in the brain, and less in gall bladder, female gonad and pancreas.

### *In Vitro* Activation by xSCT on *R. rugulosa* Pancreatic Ductal Cells

**Figure 5:**
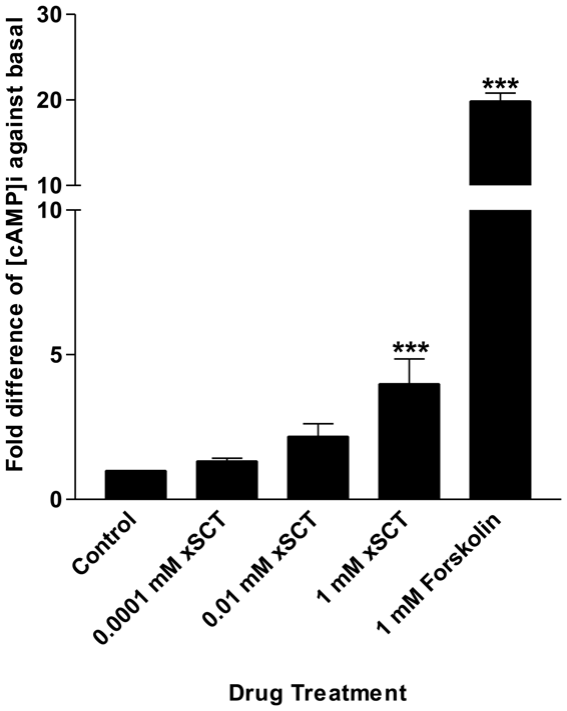
Effects of xSCT on cAMP production in primary culture of *R. rugulosa* pancreatic ductal cells. Graded concentrations of xSCT dose-dependently stimulated the [cAMP]_i_ in cultured pancreatic ductal cells. Forskolin was used in each experiment as a positive control to show the viability of the pancreatic ductal cells. Data are expressed as mean ± SEM (n = 4, *** indicates *P*<0.001).

The function of mammalian secretin in stimulating pancreatic secretion is well-established. In our study, we have therefore tested the function of xSCT by monitoring *in vitro* cAMP stimulation using primary pancreatic ductal cell culture prepared from *R. rugulosa* (Fig. 5). As an indicator of cell viability upon overnight culture, sealing of both ends of the pancreatic ducts were observed under microscope. xSCT was found to dose-dependently stimulate cAMP production; thus, affirming its effect on activating pancreatic secretion in frogs. Forskolin was used in each independent trial as a positive control and was able to induce a 20-fold increase in cAMP level when compared with the basal.

### Reciprocal Activation of Secretin and Orexin Receptors by their Mutual Endogenous Ligands

**Figure 6:**
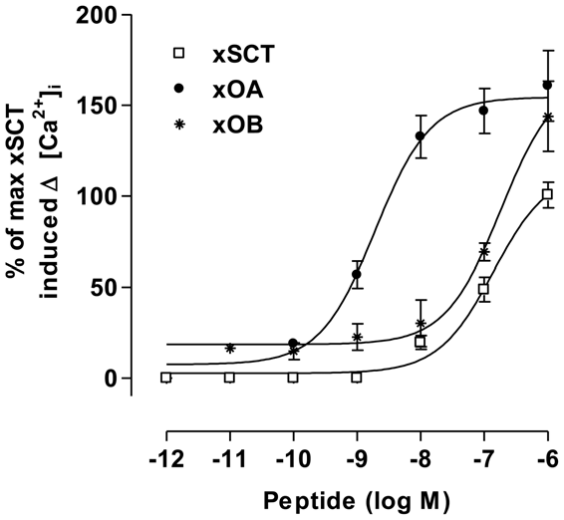
Effects of *X. laevis* secretin and orexin peptides on [Ca^2+^]_i_ in xOX2R-CHO cells. Data are expressed as ΔRFU value (maximum changes in the fluorescence signals from baseline) and converted to percentage of maximum xSCT-induced [Ca^2+^]_i_ increase. Results are expressed as mean ± SEM from 10 independent experiments, cell number = 20 to 50.

Since SCT was found only in frogs but not in lungfish, we are thus limited to testing the reciprocal activation of SCT and orexin with their receptors in frogs. In this study, we cloned the first amphibian orexin type-2 receptor from *X. laevis* (xOX2R, cDNA GenBank accession no. HQ242647) (Fig. S9). Type-2 orexin receptor was cloned because it is the only orexin receptor present and has previously been characterized in non-mammalian vertebrates. xOX2R shares a high level of sequence identity (80%) and structural similarity with its mammalian orthologs. lfSCTR, xSCTR and xOX2R were transiently expressed and exposed to graded concentrations of *Xenopus* orexins and secretin followed by measurements of intracellular cAMP accumulation and calcium mobilization. Interestingly, xOA, but not xOB, could increase dose-dependently calcium level in both lfSCTR- (EC_50_ = 1.2 nM) and xSCTR- (EC_50_ = 8.6 nM) transfected cells (Fig. 3E and F respectively). In xOX2R-expressing cells, orexins (xOA and xOB) and xSCT could trigger dose-dependent [Ca^2+^]_i_ elevations, but not intracellular cAMP accumulation, with EC_50_ values at 2.0 nM, 215 nM, and 146 nM, respectively (Fig. 6 and S10). Other peptides of the secretin/glucagon superfamily failed to elicit any responses in both cAMP and calcium assays (data not shown). These data suggest that calcium mobilization instead of cAMP is used as the main signaling pathway for orexin receptors from amphibians to mammals. Human orexin A and B were also able to induce calcium elevations in xOX2R-CHO cells, but not in SCTR-expressing cells (data not shown).

## Discussion

### Structural and Functional Evolution of SCTR in Vertebrates

To examine the origin of secretin receptor, previously known only from mammals, we tried to clone orthologs from more distantly related species – frog (*X. laevis*) and lungfish (*P. dolloi*). We identified orthologs (xSCTR and lfSCTR), indicating that this receptor originated much earlier than previously thought. Its cognate ligand, secretin, was only found in *X. laevis* but not in lungfish. Despite repeated trials on varying conditions and different designs of degenerate primers, we were not able to amplify a secretin-like sequence in lungfish. As the same PCR-based approach was adopted for the molecular cloning of secretin in frog and lungfish, we evaluated the failure in lungfish was probably attributed to the absence of secretin. Because the genomes of lungfish and other lobe-finned fish are not available, we tried to search for secretin-like sequences in other fish genomes (fugu, medaka, zebrafish, tetraodon, and stickleback). Again, secretin-like sequences were not found (data not shown). Substantiated by these evidences, we proposed that secretin does not exist in fish.

As shown by functional assays, both xSCTR and lfSCTR were coupled to cAMP and calcium signaling pathways albeit differential ligand affinities. Our data indicated that xSCTR is highly selective for frog SCT over other secretin-related peptides tested. On the other hand, it is interesting to note that hVIP and hPACAP were consistently more specific than hSCT and xSCT in stimulating lfSCTR in both cAMP and calcium pathways. Since PACAP and VIP are highly conserved peptides in vertebrates, and since we are unable to find secretin sequences in fish, we therefore hypothesize here that although the lungfish SCTR is structurally more similar to SCTRs in vertebrates, it functions as a VIP/PACAP receptor in this fish model.

Based on these findings and topology of the phylogenetic tree, we postulate that SCTR was descended from a VPAC-like receptor early in the vertebrate lineage (Figure 1A). Suggested by its newly discovered role in water homeostasis in mammals [48], [49], it is possible that the occurrence of SCTR in the Sarcopterygii lineage prior the emergence of tetrapods was used for the change from aquatic to terrestrial habitat. Due to its origin, the lfSCTR is still able to interact with and be activated by VIP and PACAP, possibly by retaining the VIP/PACAP recognition motifs (e.g. IIRIL and PDI/V). The SCTR then co-evolved with SCT leading to the divergence of functional SCT/SCTR axis paralleled with the emergence of amphibians. Descended from lobe-finned fish, the SCTR-like sequence gradually increased its specificity and sensitivity towards the newly appeared SCT, until it became fully functional as a specific receptor for SCT; and as a result, functions of VIP/PACAP and SCT were independently regulated in tetrapods.

Although we did not identify a SCT-like sequence in lungfish, we cannot exclude the possibility of the presence of a SCT-like peptide in lungfish or other lobe-finned fish species (e.g. coelacanth). If a SCT-like peptide does exist before the divergence of amphibians, it suggests that the fish secretin could have contributed to the modeling of SCTR's ligand specificity in direction to establish a functional SCT-SCTR axis in amphibians.

### Cross-Interactions of SCT and Orexin with their Receptors in Frog

The question of whether secretin and orexin as well as their receptors shared the same origin early in the vertebrate lineage is elusive and was a controversial issue in the past. In the present study, with the cloning of orexin type-2 receptor from *X. laevis*, we demonstrated that, in frog, orexin and secretin could reciprocally activate their receptors. Of all the secretin/glucagon superfamily peptides, the ability of stimulating orexin receptor is, however, limited to secretin. Hence, it is unlikely that orexin and secretin are sharing a common ancestral origin since the superfamily peptides are well documented for their cross reactivity towards each other's receptors [50]–[52]. Structurally, frog secretin and orexin share moderate sequence homology (43%) when the N-terminus of SCT (HAAGILT) is compared to the C-terminus of OA (HVDGRFT). As the N-terminus of SCT is crucial for receptor binding, the observed structural similarity shared by these peptides, likely due to convergent evolution, may explain their cross reactivity with respective receptors.

The interesting reciprocal ligand-receptor activation exhibited hereby suggests that secretin and orexins were complementary partners in mediating similar and/or overlapping physiological processes via stimulating SCTR and OX2R in non-mammalian vertebrates of the Sarcopterygii lineage. Taking together the ability of orexin in activating lfSCTR, we postulate a major alteration of ligand-receptor interaction of secretin and orexin by the advent of mammals. Before the divergence of mammals, SCT and orexin A were in coordination in activating SCTR and OX2R; while orexin B is specific to activating OX2R to mediate functions distinct from those controlled by SCT and orexin A. By the divergence of mammals, orexin type-1 receptor (OX1R) emerged to facilitate the precise control over the biological functions mediated by orexin receptors. OX2R in mammals retained its ligand specificity for orexin A and B descended along the vertebrate lineage; while OX1R is specifically activated by orexin A. The functional diversifications of orexin receptors and SCTRs in mammals were fine-tuned to be activated by their own endogenous peptides, resulting in the loss of the reciprocal activation observed in non-mammalian vertebrates.

Though secretin and orexin evolved independently, their functions converged at some point in the vertebrate lineage. This convergent evolutionary pattern is, however, not limited to secretin and orexin. For instance, the intraflagellar transport (IFT) genes and Regulatory Factor X transcription factors (RFX TFs) have recently been reported to have evolved independently in pre-metazoans, and their evolution converged to establish a transcriptional regulatory relationship in metazoans [53]. It was suggested that the convergent molecular evolution of IFT genes and RFX TFs could have provided a pivotal driving force in the evolution and emergence of metazoans [53]. In the case of secretin and orexin, their convergence was likely the result of selection by similar adaptive pressures [54], [55]. Their complementary mediation on crucial biological functions could have driven the establishment of enhanced and networked control for the adaptation of amphibians in both terrestrial and aquatic habitats. Analogy of secretin and orexin could have been established by the neutral drift of molecular adaptation, in which the neutral emergence of non-specific binding was one of the possible mechanisms.

Integration of our current findings, we conclude that secretin receptors are not exclusively expressed and functional in mammals, thus are not encoded from gene(s) that were duplicated or modified along with the speciation of mammals. Their tissue-specific expression and abilities in triggering classical GPCR signaling pathways affirmed that they were already physiologically functional prior mammalian divergence, and are most likely descended from a VPAC-like receptor prior the sarcopterygian-actinopterygian split that occurred after the second round of whole genome duplication. We also showed that, despite the reciprocal activation of secretin and orexin receptor by their mutual endogenous ligands in non-mammalian vertebrates, secretin and orexin are of different ancestral origins early in the vertebrate lineage. We speculate that the analogy observed in secretin and orexin was a result of convergent evolution, in which their cross-reactivity could be established by the neutral emergence of non-specific binding.

## Materials and Methods

### Ethics Statement

All animal treatments were in accordance with the guidelines established by the Committee on the Use of Live Animals in Teaching and Research (CULATR, Approval ID 1496-07) of the University of Hong Kong with the Cap. 340 animal license issued by the Department of Health of the Hong Kong Government under the Animals Ordinance.

### Animals and Peptides

Lungfish *P. dolloi* and frog *R. rugulosa* were purchased from a local commercial supplier, and *X. laevis* was bought from Xenopus I (Xenopus I, Inc., CA). Glucagon, glucagon-like peptides, GIP, GHRH, PRP, PACAP peptides were ordered from the Proteomics Resource Center of the Rockefeller University (http://proteomics.rockefeller.edu/). *Xenopus* orexin A and B were ordered from Shanghai HanHong Chemical (Shanghai HanHong Chemical Co., Ltd., Shanghai, China). VIP and PHI peptides were synthesized by Bachem California (Bachem California, Inc., CA). Human SCT was bought from AnaSpec (AnaSpec, Inc., CA), and xSCT was synthesized by one of us (Alain Fournier). All synthetic peptides were of >95.0% purity.

### Total RNA Extraction and First-strand cDNA Synthesis

Animals were sacrificed by cervical decapitation. Total RNA was isolated from freshly excised tissues by TriPure reagent according to the manufacturer's instructions (Invitrogen, Carlsbad, CA). First-strand cDNA from 5 µg total RNA was synthesized according to the protocol of SuperScript III Reverse Transcriptase (Invitrogen) using the Adaptor Primer (AP) (Invitrogen).

### Molecular Cloning of Frog Secretins, Secretin Receptors from *P. dolloi* and *X. laevis*, and *X. laevis* Orexin Type-2 Receptor

Primers for the amplification of *X. laevis* SCT (xSCT), SCTR (xSCTR), and orexin type-2 receptor (xOX2R) and *R. rugulosa* SCT (Rana SCT) were designed based on the partial sequences obtained from the *X. tropicalis* genome by a BLAST search. Degenerate primers for the amplification of lungfish SCTR (lfSCTR) were designed according to conserved regions of aligned SCTR sequences (Primer List, Table S1). Rapid amplification of cDNA ends (RACE) was performed using the 5′ and 3′ RACE amplification kits (Invitrogen) with specific primers designed according to the partial sequences. Full-length cDNA clones encompassing the 5′ to 3′ untranslated regions were produced by PCR with specific primers and confirmed by DNA sequencing. Full-length SCTR cDNAs were subcloned to pcDNA3.1 (+) (Invitrogen) for functional expression. All sequences newly identified in the present study have been deposited in the GenBank (Genbank accession no.: xSCT, HQ236553; xSCTR, HQ236552; xOX2R, HQ242647; Rana SCT, HQ236554; lfSCTR, HQ236551).

### Tissue Distribution of Secretin and Secretin Receptor in *P. dolloi* and *X. laevis*

Quantitative real-time PCR was used to determine the expression profiles of SCT and SCTR in *P. dolloi* and *X. laevis* in various tissues. First-strand cDNAs were synthesized from total RNA as previously mentioned (Materials and Methods: Total RNA Extraction and First-strand cDNA Synthesis). RT-PCR (n = 4, each in duplicates) was performed using the Power SYBR Green PCR Master Mix (Applied Biosystems, Foster City, CA) and the 7300 Real Time PCR System (Applied Biosystems). Primers used in the real-time PCR are listed in Table S1. The threshold cycle (Ct) is defined as the fractional cycle number at which the fluorescence reaches 10-fold standard deviation of the baseline (from cycle 3 to 10). The specificity of the SYBR PCR signal was confirmed by both melt curve analysis and agarose gel electrophoresis. Standard curves were established by 10× serial dilution of respective plasmid stocks.

### Transient Expression of Secretin and Orexin Receptors in CHO cells

Chinese Hamster Ovary (CHO) cells (ATCC, Manassas, VA) were cultured in MEM/10% FBS/100 U/ml Penicillin/100 g/ml Streptomycin on 100 mm tissue culture plates at 37°C and 5% CO_2_ until 80% confluence. SCTR or OX2R expression construct (2 µg) was used to transfect 1×10^5^ CHO cells with 6 µl GeneJuice reagent (Novagen, Darmstadt, Germany). A control cell line was established by transfecting the cells with the pcDNA 3.1 (+) vector (Invitrogen). Intracellular cAMP production upon peptide stimulation was measured using the LANCE cAMP assay kit (Perkin-Elmer, Waltham, MA) in the Victor ×4 multilabel reader (Perkin-Elmer) according to the manufacturer's protocol. Intracellular cAMP levels ([cAMP]_i_) were measured and expressed as cAMP concentration relative to the basal level (stimulation buffer alone without peptide addition). Negative control experiments were performed by simultaneous peptide stimulation at 10 µM on the control cell line in each experimental trial.

For confocal calcium imaging, transiently transfected cells were plated at a density of 3000 cells/well in 24-well plates (Sigma-Aldrich, St. Louis, MO). After overnight incubation, cells were pre-loaded with 5 µM Fluo-3 acetoxymethyl ester (AM) (Sigma) for 45 min at 37°C in Tyrode solution consisting of (mM): 140 NaCl, 5 KCl, 1 MgCl_2_, 1 CaCl_2_, 10 glucose and 10 HEPES at pH 7.4. Calcium transient of single receptor-transfected CHO cell was recorded with a confocal imaging system (Olympus Fluoview System version 4.2 FV300 TIEMPO) mounted on an upright Olympus microscope (IX71). Peptides at concentrations ranging from 10^−5^ to 10^−12^ M were added at designated time point and calcium level was traced in a real-time manner using the Fluoview software (Olympus). Data were expressed in ΔRFU value (maximum changes in the fluorescence signals from baseline) and converted to percentage of the maximum of xSCT-induced [Ca^2+^]_i_ elevation (i.e. xSCT [Ca^2+^]_i_ at 10 µM = 100%). For both assays, ionomycin (10 µM) was added at the end of each experiment to test the vitality of the cells.

### *In Vitro* Activation by xSCT on *R. rugulosa* Pancreatic Ductal Cells in Primary Culture

Adult male *R. rugulosa* was sacrificed by cervical decapitation. The freshly excised pancreas was removed and washed in Ringer solution (in mM, 85 NaCl, 4 KCl, 17.5 NaHCO_3_, 0.8 KH_2_PO_4_, 2 glucose, 1.5 CaCl_2_ and 0.8 MgCl_2_, pH 7.6). The excised pancreas was then minced into approximately 1 mm^3^ pieces and digested at room temperature for 1 hr in the dissociation medium (Ringer solution supplemented with 0.2 mg/ml soy beans trypsin inhibitor (Sigma), 2 mg/ml BSA (Roche), 400 U/ml hyaluronidase (Sigma) and 50 U/ml collagenase (Sigma)). The tissue suspension was then washed with Ringer solution and small clusters of dissociated cells were filtered with the use of a 100-µm cell strainer (BD Falcon, US). Filtrate containing the undigested tissue, including the pancreatic ducts, was maintained in culture medium (80% Leibovitz's L-15 medium (Invitrogen) supplemented with 10% FBS and 100 U/ml Penicillin/100 g/ml Streptomycin) in a humidified incubator at 24°C. Upon overnight culture, both ends of the pancreatic ducts sealed with the lumens dilated due to accumulation of the secretion as seen under microscope. The isolated pancreatic ductal cells were then used for cAMP stimulation in the presence of xSCT peptides and forskolin.

### Phylogenetic Analysis

Amino acid sequences were aligned with Clustal X, and phylogenetic trees were constructed using MEGA 5.0 software [56]. The best-fit models of the trees were selected by ProtTest 3.0 [57]. The trees were calculated by Maximum Likelihood method with the Jones-Taylor-Thornton (JTT) model and combined with +I: invariable sites, +G: rate heterogeneity among sites, +F: observed amino acid frequencies. 1000 bootstrap simulations were used to test the reliability of branching. Numbers on the nodes of the tree indicated the percentage of bootstrap replicates in which the labeled branch was reproduced. Sequences used in these analyses and their accession numbers retrieved from Genbank and Ensembl are shown in Table S2.

### Statistical Analysis

Results are presented as mean ± SEM, and are averages of the means of duplicated assays in at least three independent experiments. GraphPad Prism version 3.0 (GraphPad Software, Inc., San Diego, CA) was used to plot the sigmoidal curves in the cAMP and calcium mobilization assays and to perform statistical analyses using one-way ANOVA followed by Dunnett's test. Differences were considered significant when *P*<0.05.

## Supporting Information

Figure S1 Nucleotide (GenBank accession no. HQ236552) and deduced amino acid sequence of the *X. laevis* secretin receptor (xSCTR) cDNA. Nucleotides (lower line) and amino acids (upper line) are numbered from the initiation methionine. The signal peptide (24 amino acids) is indicated in bold characters. Transmembrane domains are underlined with solid lines.
(PPTX)

Figure S2 Nucleotide (GenBank accession no. HQ236551) and deduced amino acid sequence of the *P. dolloi* secretin receptor (lfSCTR) cDNA. Nucleotides (lower line) and amino acids (upper line) are numbered from the initiation methionine residue. The signal peptide (28 amino acids) is indicated in bold characters. Transmembrane domains are underlined with solid lines.
(PPTX)

Figure S3 Percent amino acid homology of vertebrate secretin receptor (A) ligand-binding domain and (B) entire sequence.
(PPTX)

Figure S4 Alignment of cloned secretin receptor amino acid sequences. Putative transmembrane domains are overlined and labeled. # and * indicate potential sites for N-linked glycosylation and conserved cysteine residues, respectively. Predicted ligand-binding domains are indicated in bold characters. Structural features are boxed with dotted lines. Gaps (represented by - ) were introduced to maximize sequence homology.
(PPTX)

Figure S5 Nucleotide (GenBank accession no. HQ236553) and deduced amino acid sequence of the *X. laevis* secretin (xSCT) cDNA. The full-length xSCT is 2141 bp in length. Nucleotides (lower line) and amino acids (upper line) are numbered from the initiation methionine residue. The signal peptide (20 amino acids) is indicated in bold characters. The mature peptide (28 amino acids) is underlined with solid line and the potential cleavage/amidation site (GKR) is boxed.
(PPTX)

Figure S6 Nucleotide (GenBank accession no. HQ236554) and deduced amino acid sequence of the *R. rugulosa* secretin (Rana SCT) cDNA. The full-length Rana SCT is 763 bp in length. Nucleotides (lower line) and amino acids (upper line) are numbered from the initiation methionine residue. The signal peptide (22 amino acids) is indicated in bold characters. The mature peptide (28 amino acids) is underlined with solid line and the potential cleavage/amidation site (GKR) is boxed.
(PPTX)

Figure S7 Sequence analyses of secretin mature peptides. (A) Alignment of secretin mature peptide sequences. Identical residues are indicated in bold characters. Accession numbers are: *H. sapiens*, AAG31443; *R. norvegicus*, AAA42128; *M. musculus*, CAA51982; *B. taurus*, P63296; *C. familiaris*, P09910; *O. cuniculus*, P32647; *S. scrofa*, AAA31121; *G. gallus*, NP_001020004. (B) Percent amino acid homology of the aligned secretin mature peptides.
(PPTX)

Figure S8 Representative traces of hPACAP27, hSCT, hVIP, xOA, and xSCT on intracellular calcium mobilization in lfSCTR, xSCTR and null pcDNA 3.1-transfected CHO cells. Peak magnitude of traces is proportional to the order of potency of the ligands tested. Traces were obtained from at least 10 calcium assays with respective control shown on the right panel. Ionomycin (10 µM) was added at the end of each experiment to test the vitality of the cells.
(PPTX)

Figure S9 Full-length nucleotide (GenBank accession no. HQ242647) and deduced amino acid sequence of *X. laevis* orexin type-2 receptor (xOX2R). Numbers on the left and right indicate the position of the first nucleotide and the last amino acid of each line from the start codon, respectively. The ORF sequence is presented in upper cases whereas the 3′ and 5′ UTR sequences are presented in lower cases. The amino acid sequences corresponding to the seven putative transmembrane domains (TM1-7) are labeled and underlined. The stop codon is marked by an asterisk sign (*).
(PPTX)

Figure S10 Representative traces of xSCT, xOA, and xOB on intracellular calcium mobilization in xOX2R- and null pcDNA 3.1-transfected CHO cells. Peak magnitude of traces is proportional to the order of potency of the ligands tested. Traces were obtained from at least 10 calcium assays with respective control shown on the right panel. Ionomycin (10 µM) was added at the end of each experiment to test the vitality of the cells.
(PPTX)

Table S1 List of primers used in PCR and real-time PCR amplifications.
(PPTX)

Table S2 Accession numbers of amino acid sequences of secretin/glucagon superfamily hormones and GPCR secretin family receptors [58], [59].
(PPTX)

## References

[1] Ng SS, Yung WH, Chow BK (2002). Secretin as a neuropeptide.. Mol Neurobiol.

[2] Campbell RM, Scanes CG (1992). Evolution of the growth hormone-releasing factor (GRF) family of peptides.. Growth Regul.

[3] Vaudry D, Gonzalez BJ, Basille M, Yon L, Fournier A (2000). Pituitary adenylate cyclase-activating polypeptide and its receptors: from structure to functions.. Pharmacol Rev.

[4] Bayliss WM, Starling EH (1902). The mechanism of pancreatic secretion.. J Physiol.

[5] Ishihara T, Nakamura S, Kaziro Y, Takahashi T, Takahashi K (1991). Molecular cloning and expression of a cDNA encoding the secretin receptor.. EMBO J.

[6] Segre GV, Goldring SR (1993). Receptors for secretin, calcitonin, parathyroid hormone (PTH)/PTH-related peptide, vasoactive intestinal peptide, glucagonlike peptide 1, growth hormone-releasing hormone, and glucagon belong to a newly discovered G-protein-linked receptor family.. Trends Endocrinol Metab.

[7] Ulrich CD, Holtmann M, Miller LJ (1998). Secretin and vasoactive intestinal peptide receptors: members of a unique family of G protein-coupled receptors.. Gastroenterology.

[8] Harmar AJ (2001). Family-B G-protein-coupled receptors.. Genome Biol.

[9] Siu FK, Lam IP, Chu JY, Chow BK (2006). Signaling mechanisms of secretin receptor.. Regul Pept.

[10] Chow BK (1995). Molecular cloning and functional characterization of a human secretin receptor.. Biochem Biophys Res Commun.

[11] Jiang S, Ulrich C (1995). Molecular cloning and functional expression of a human pancreatic secretin receptor.. Biochem Biophys Res Commun.

[12] Patel DR, Kong Y, Sreedharan SP (1995). Molecular cloning and expression of a human secretin receptor.. Mol Pharmacol.

[13] Vassilatis DK, Hohmann JG, Zeng H, Li F, Ranchalis JE (2003). The G protein-coupled receptor repertoires of human and mouse.. Proc Natl Acad Sci U S A.

[14] Strausberg RL, Feingold EA, Grouse LH, Derge JG, Klausner RD (2002). Generation and initial analysis of more than 15,000 full-length human and mouse cDNA sequences.. Proc Natl Acad Sci U S A.

[15] Meuth-Metzinger VL, Philouze-Rome V, Metzinger L, Gespach C, Guilloteau P (2005). Differential activation of adenylate cyclase by secretin and VIP receptors in the calf pancreas.. Pancreas.

[16] Svoboda M, Tastenoy M, De NP, Delporte C, Waelbroeck M (1998). Molecular cloning and in vitro properties of the recombinant rabbit secretin receptor.. Peptides.

[17] Sakurai T, Amemiya A, Ishii M, Matsuzaki I, Chemelli RM (1998). Orexins and orexin receptors: a family of hypothalamic neuropeptides and G protein-coupled receptors that regulate feeding behavior.. Cell.

[18] De Lecea L, Kilduff TS, Peyron C, Gao X, Foye PE (1998). The hypocretins: hypothalamus-specific peptides with neuroexcitatory activity.. Proc Natl Acad Sci U S A.

[19] Alvarez CE, Sutcliffe JG (2002). Hypocretin is an early member of the incretin gene family.. Neurosci Lett.

[20] Kane JK, Tanaka H, Parker SL, Yanagisawa M, Li MD (2000). Sensitivity of orexin-A binding to phospholipase C inhibitors, neuropeptide Y, and secretin.. Biochem Biophys Res Commun.

[21] Holmqvist T, Akerman KE, Kukkonen JP (2001). High specificity of human orexin receptors for orexins over neuropeptide Y and other neuropeptides.. Neurosci Lett.

[22] Smart D, Sabido-David C, Brough SJ, Jewitt F, Johns A (2001). SB-334867-A: the first selective orexin-1 receptor antagonist.. Br J Pharmacol.

[23] Panula P (2010). Hypocretin/orexin in fish physiology with emphasis on zebrafish.. Acta Physiol (Oxf).

[24] Galas L, Vaudry H, Braun B, Van Den Pol AN, De Lecea L (2001). Immunohistochemical localization and biochemical characterization of hypocretin/orexin-related peptides in the central nervous system of the frog Rana ridibunda.. J Comp Neurol.

[25] Ohkubo T, Boswell T, Lumineau S (2002). Molecular cloning of chicken prepro-orexin cDNA and preferential expression in the chicken hypothalamus.. Biochim Biophys Acta.

[26] Ohkubo T, Tsukada A, Shamoto K (2003). cDNA cloning of chicken orexin receptor and tissue distribution: sexually dimorphic expression in chicken gonads.. J Mol Endocrinol.

[27] Feillet CA (2010). Food for thoughts: feeding time and hormonal secretion.. J Neuroendocrinol.

[28] Tomasik PJ, Sztefko K (2009). The effect of enteral and parenteral feeding on secretion of orexigenic peptides in infants.. BMC Gastroenterol.

[29] Silva JP, von Meyenn F, Howell J, Thorens B, Wolfrum C (2009). Regulation of adaptive behaviour during fasting by hypothalamic Foxa2.. Nature.

[30] Bulbul M, Tan R, Gemici B, Ozdem S, Ustunel I (2010). Endogenous orexin-A modulates gastric motility by peripheral mechanisms in rats.. Peptides.

[31] Szlachcic A, Brzozowski T, Majka J, Pajdo R, Konturek PC (2010). Involvement of Orexigenic Peptides in the Mechanism of Gastric Mucosal Integrity and Healing of Chronic Gastric Ulcers.. Curr Pharm Des.

[32] Flemstrom G, Bengtsson MW, Makela K, Herzig KH (2010). Effects of short-term food deprivation on orexin-A-induced intestinal bicarbonate secretion in comparison with related secretagogues.. Acta Physiol (Oxf).

[33] Tsujino N, Sakurai T (2009). Orexin/hypocretin: a neuropeptide at the interface of sleep, energy homeostasis, and reward system.. Pharmacol Rev.

[34] Heinonen MV, Purhonen AK, Makela KA, Herzig KH (2008). Functions of orexins in peripheral tissues.. Acta Physiol (Oxf).

[35] Sutcliffe JG, De Lecea L (1999). Novel neurotransmitters for sleep and energy homeostasis.. Results Probl Cell Differ.

[36] Nilsson A, Carlquist M, Jornvall H, Mutt V (1980). Isolation and characterization of chicken secretin.. Eur J Biochem.

[37] Cardoso JC, Vieira FA, Gomes AS, Power DM (2010). The serendipitous origin of chordate secretin peptide family members.. BMC Evol Biol.

[38] Cardoso JC, Pinto VC, Vieira FA, Clark MS, Power DM (2006). Evolution of secretin family GPCR members in the metazoa.. BMC Evol Biol.

[39] Bailes HJ, Trezise AE, Collin SP (2007). The optics of the growing lungfish eye: lens shape, focal ratio and pupillary movements in Neoceratodus forsteri (Krefft, 1870).. Vis Neurosci.

[40] Zardoya R, Abouheif E, Meyer A (1996). Evolutionary analyses of hedgehog and Hoxd-10 genes in fish species closely related to the zebrafish.. Proc Natl Acad Sci U S A.

[41] San Mauro D (2010). A multilocus timescale for the origin of extant amphibians.. Mol Phylogenet Evol.

[42] Graham JB, Lee HJ (2004). Breathing air in air: in what ways might extant amphibious fish biology relate to prevailing concepts about early tetrapods, the evolution of vertebrate air breathing, and the vertebrate land transition?. Physiol Biochem Zool.

[43] Bockaert J, Pin JP (1999). Molecular tinkering of G protein-coupled receptors: an evolutionary success.. EMBO J.

[44] Grace CR, Perrin MH, DiGruccio MR, Miller CL, Rivier JE (2004). NMR structure and peptide hormone binding site of the first extracellular domain of a type B1 G protein-coupled receptor.. Proc Natl Acad Sci U S A.

[45] Miller LJ, Dong M, Harikumar KG, Gao F (2007). Structural basis of natural ligand binding and activation of the Class II G-protein-coupled secretin receptor.. Biochem Soc Trans.

[46] Chan KY, Pang RT, Chow BK (2001). Functional segregation of the highly conserved basic motifs within the third endoloop of the human secretin receptor.. Endocrinology.

[47] Fradinger EA, Tello JA, Rivier JE, Sherwood NM (2005). Characterization of four receptor cDNAs: PAC1, VPAC1, a novel PAC1 and a partial GHRH in zebrafish.. Mol Cell Endocrinol.

[48] Chu JY, Lee LT, Lai CH, Vaudry H, Chan YS (2009). Secretin as a neurohypophysial factor regulating body water homeostasis.. Proc Natl Acad Sci U S A.

[49] Cheng CY, Chu JY, Chow BK (2009). Vasopressin-independent mechanisms in controlling water homeostasis.. J Mol Endocrinol.

[50] Van Rampelbergh J, Gourlet P, De Neef P, Robberecht P, Waelbroeck M (1996). Properties of the pituitary adenylate cyclase-activating polypeptide I and II receptors, vasoactive intestinal peptide1, and chimeric amino-terminal pituitary adenylate cyclase-activating polypeptide/vasoactive intestinal peptide1 receptors: evidence for multiple receptor states.. Mol Pharmacol.

[51] Perret J, Van Craenenbroeck M, Langer I, Vertongen P, Gregoire F (2002). Mutational analysis of the glucagon receptor: similarities with the vasoactive intestinal peptide (VIP)/pituitary adenylate cyclase-activating peptide (PACAP)/secretin receptors for recognition of the ligand's third residue.. Biochem J.

[52] Laburthe M, Couvineau A, Marie JC (2002). VPAC receptors for VIP and PACAP.. Receptors Channels.

[53] Chu JS, Baillie DL, Chen N (2010). Convergent evolution of RFX transcription factors and ciliary genes predated the origin of metazoans.. BMC Evol Biol.

[54] Castoe TA, de Koning AP, Kim HM, Gu W, Noonan BP (2009). Evidence for an ancient adaptive episode of convergent molecular evolution.. Proc Natl Acad Sci U S A.

[55] Massey SE, Churbanov A, Rastogi S, Liberles DA (2008). Characterizing positive and negative selection and their phylogenetic effects.. Gene.

[56] Tamura K, Dudley J, Nei M, Kumar S (2007). MEGA4: Molecular Evolutionary Genetics Analysis (MEGA) software version 4.0.. Mol Biol Evol.

[57] Abascal F, Zardoya R, Posada D (2005). ProtTest: selection of best-fit models of protein evolution.. Bioinformatics.

[58] Hoo RL, Alexandre D, Chan SM, Anouar Y, Pang RT (2001). Structural and functional identification of the pituitary adenylate cyclase-activating polypeptide receptor VPAC2 from the frog Rana tigrina rugulosa.. J Mol Endocrinol.

[59] Tse DL, Pang RT, Wong AO, Chan SM, Vaudry H (2002). Identification of a potential receptor for both peptide histidine isoleucine and peptide histidine valine.. Endocrinology.

