# Supplementary material for: Origin of Secretin Receptor Precedes the Advent of Tetrapoda: Evidence on the Separated Origins of Secretin and Orexin
Source: PLoS One. 2011 Apr 29;6(4):e19384. doi: 10.1371/journal.pone.0019384 (PMC3084839; doi:10.1371/journal.pone.0019384)
Supplement: Figure S1 — Nucleotide (GenBank accession no. HQ236552) and deduced amino acid sequence of the X. laevis secretin receptor (xSCTR) cDNA. Nucleotides (lower line) and amino acids (upper line) are numbered from the initiation methionine. The signal peptide (24 amino acids) is indicated in bold characters. Transmembrane domains are underlined with solid lines. (PPTX) [file pone.0019384.s001.pptx]

## Slide 1
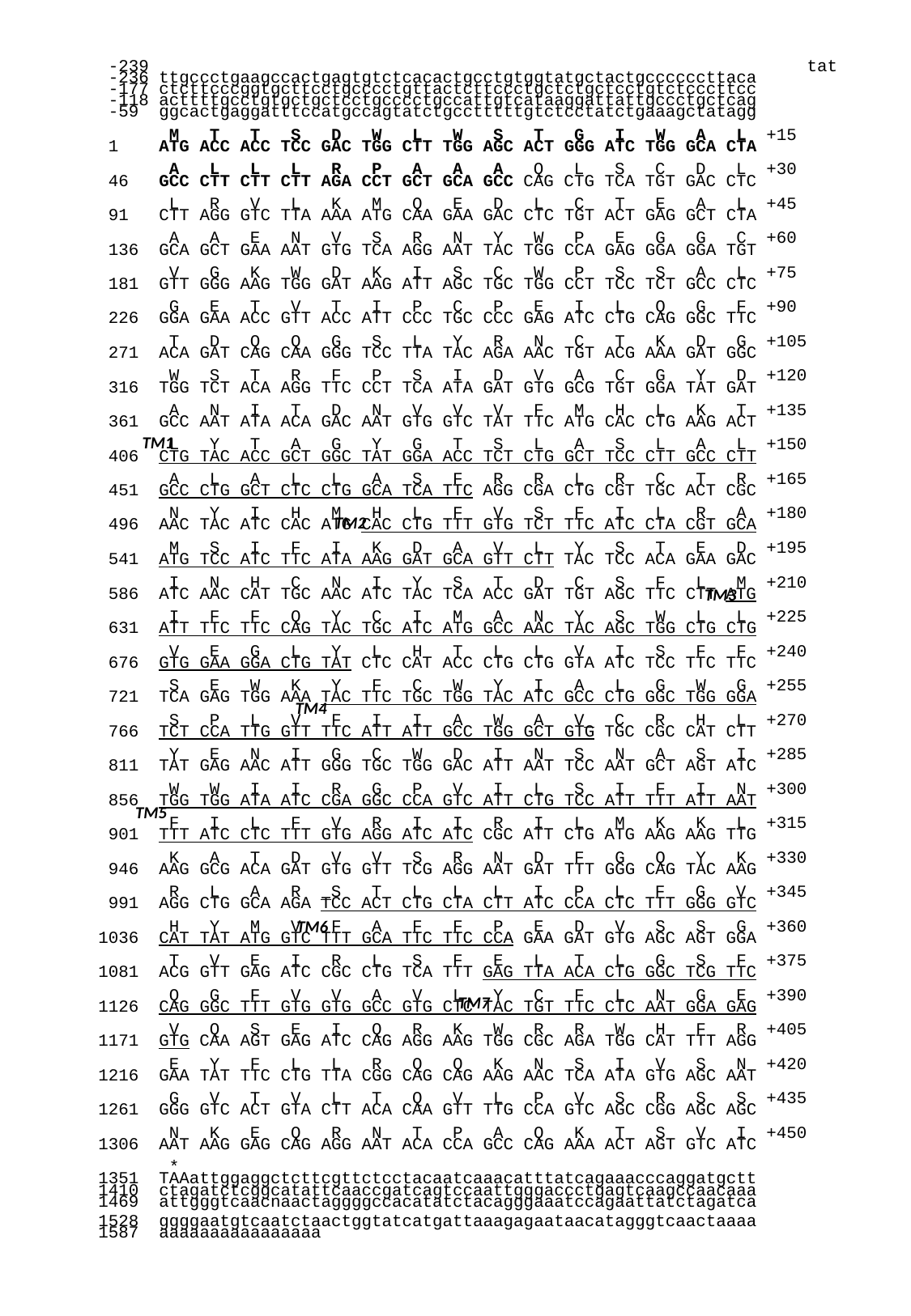

-239						 tat
 -236	ttgccctgaagccactgagtgtctcacactgcctgtggtatgctactgccccccttaca
 -177	ctcttcccggtgcttcctgcccctgttactcttccctgctctgctcctgtctcccttcc
 -118	acttttgcctgtgctgctcctgcccctgccattgtcataaggattattgccctgctcag
 -59	ggcactgaggatttccatgccagtatctgcctttttgtctcctatctgaaagctatagg
	 M T T S D W L W S T G I W A L	+15
 1	ATG ACC ACC TCC GAC TGG CTT TGG AGC ACT GGG ATC TGG GCA CTA
 A L L L R P A A A Q L S C D L 	+30
 46	GCC CTT CTT CTT AGA CCT GCT GCA GCC CAG CTG TCA TGT GAC CTC
	 L R V L K M Q E D L C T E A L	+45
 91	CTT AGG GTC TTA AAA ATG CAA GAA GAC CTC TGT ACT GAG GCT CTA
	 A A E N V S R N Y W P E G G C	+60
 136	GCA GCT GAA AAT GTG TCA AGG AAT TAC TGG CCA GAG GGA GGA TGT
	 V G K W D K I S C W P S S A L	+75
 181	GTT GGG AAG TGG GAT AAG ATT AGC TGC TGG CCT TCC TCT GCC CTC
	 G E T V T I P C P E I L Q G F 	+90
 226	GGA GAA ACC GTT ACC ATT CCC TGC CCC GAG ATC CTG CAG GGC TTC
	 T D Q Q G S L Y R N C T K D G 	+105
 271	ACA GAT CAG CAA GGG TCC TTA TAC AGA AAC TGT ACG AAA GAT GGC
	 W S T R F P S I D V A C G Y D	+120
 316	TGG TCT ACA AGG TTC CCT TCA ATA GAT GTG GCG TGT GGA TAT GAT
	 A N I T D N V V V F M H L K T 	+135
 361	GCC AAT ATA ACA GAC AAT GTG GTC TAT TTC ATG CAC CTG AAG ACT
	 L Y T A G Y G T S L A S L A L	+150
 406	CTG TAC ACC GCT GGC TAT GGA ACC TCT CTG GCT TCC CTT GCC CTT
	 A L A L L A S F R R L R C T R	+165
 451	GCC CTG GCT CTC CTG GCA TCA TTC AGG CGA CTG CGT TGC ACT CGC
	 N Y I H M H L F V S F I L R A	+180
 496	AAC TAC ATC CAC ATG CAC CTG TTT GTG TCT TTC ATC CTA CGT GCA
	 M S I F I K D A V L Y S T E D	+195
 541 ATG TCC ATC TTC ATA AAG GAT GCA GTT CTT TAC TCC ACA GAA GAC
	 I N H C N I Y S T D C S F L M	+210
 586	ATC AAC CAT TGC AAC ATC TAC TCA ACC GAT TGT AGC TTC CTT ATG
	 I F F Q Y C I M A N Y S W L L	+225
 631	ATT TTC TTC CAG TAC TGC ATC ATG GCC AAC TAC AGC TGG CTG CTG
	 V E G L Y L H T L L V I S F F	+240
 676	GTG GAA GGA CTG TAT CTC CAT ACC CTG CTG GTA ATC TCC TTC TTC
	 S E W K Y F C W Y I A L G W G 	+255
 721	TCA GAG TGG AAA TAC TTC TGC TGG TAC ATC GCC CTG GGC TGG GGA
	 S P L V F I I A W A V C R H L	+270
 766	TCT CCA TTG GTT TTC ATT ATT GCC TGG GCT GTG TGC CGC CAT CTT
	 Y E N I G C W D I N S N A S I	+285
 811	TAT GAG AAC ATT GGG TGC TGG GAC ATT AAT TCC AAT GCT AGT ATC
	 W W I I R G P V I L S I F I N	+300
 856	TGG TGG ATA ATC CGA GGC CCA GTC ATT CTG TCC ATT TTT ATT AAT
	 F I L F V R I I R I L M K K L	+315
 901	TTT ATC CTC TTT GTG AGG ATC ATC CGC ATT CTG ATG AAG AAG TTG
	 K A T D V V S R N D F G Q Y K	+330
 946 AAG GCG ACA GAT GTG GTT TCG AGG AAT GAT TTT GGG CAG TAC AAG
	 R L A R S T L L L I P L F G V	+345
 991	AGG CTG GCA AGA TCC ACT CTG CTA CTT ATC CCA CTC TTT GGG GTC
	 H Y M V F A F F P E D V S S G	+360
 1036	CAT TAT ATG GTC TTT GCA TTC TTC CCA GAA GAT GTG AGC AGT GGA
	 T V E I R L S F E L T L G S F	+375
 1081	ACG GTT GAG ATC CGC CTG TCA TTT GAG TTA ACA CTG GGC TCG TTC
	 Q G F V V A V L Y C F L N G E	+390
 1126	CAG GGC TTT GTG GTG GCC GTG CTC TAC TGT TTC CTC AAT GGA GAG
	 V Q S E I Q R K W R R W H F R 	+405
 1171	GTG CAA AGT GAG ATC CAG AGG AAG TGG CGC AGA TGG CAT TTT AGG
	 E Y F L L R Q Q K N S I V S N 	+420
 1216	GAA TAT TTC CTG TTA CGG CAG CAG AAG AAC TCA ATA GTG AGC AAT
	 G V T V L T Q V L P V S R S S 	+435
 1261	GGG GTC ACT GTA CTT ACA CAA GTT TTG CCA GTC AGC CGG AGC AGC
	 N K E Q R N T P A Q K T S V I	+450
 1306	AAT AAG GAG CAG AGG AAT ACA CCA GCC CAG AAA ACT AGT GTC ATC
	 *
 1351	TAAattggaggctcttcgttctcctacaatcaaacatttatcagaaacccaggatgctt
 1410	ctagatctcggcatattcaaccgatcagtccaattgggaccctgagtcaagccaacaaa
 1469	attgggtcaacnaactaggggccacatatctacagggaaatccagaattatctagatca
 1528	ggggaatgtcaatctaactggtatcatgattaaagagaataacatagggtcaactaaaa
 1587	aaaaaaaaaaaaaaaa
TM1
TM2
TM3
TM4
TM5
TM6
TM7
