# Supplementary material for: Origin of Secretin Receptor Precedes the Advent of Tetrapoda: Evidence on the Separated Origins of Secretin and Orexin
Source: PLoS One. 2011 Apr 29;6(4):e19384. doi: 10.1371/journal.pone.0019384 (PMC3084839; doi:10.1371/journal.pone.0019384)
Supplement: Figure S2 — Nucleotide (GenBank accession no. HQ236551) and deduced amino acid sequence of the P. dolloi secretin receptor (lfSCTR) cDNA. Nucleotides (lower line) and amino acids (upper line) are numbered from the initiation methionine residue. The signal peptide (28 amino acids) is indicated in bold characters. Transmembrane domains are underlined with solid lines. (PPTX) [file pone.0019384.s002.pptx]

## Slide 1
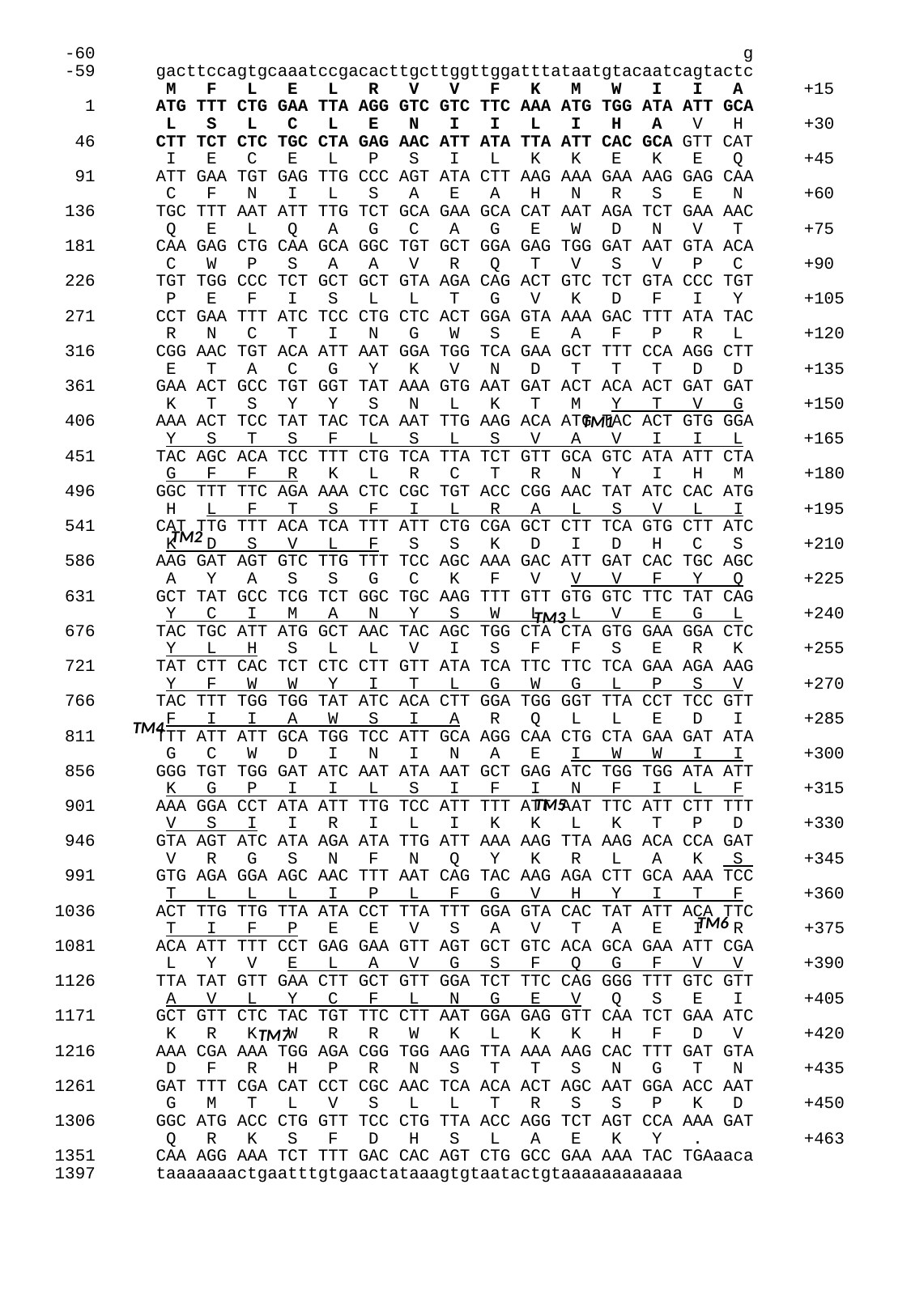

-60					 g
 -59	gacttccagtgcaaatccgacacttgcttggttggatttataatgtacaatcagtactc
	 M F L E L R V V F K M W I I A +15
 1	ATG TTT CTG GAA TTA AGG GTC GTC TTC AAA ATG TGG ATA ATT GCA
	 L S L C L E N I I L I H A V H +30
 46	CTT TCT CTC TGC CTA GAG AAC ATT ATA TTA ATT CAC GCA GTT CAT
	 I E C E L P S I L K K E K E Q +45
 91	ATT GAA TGT GAG TTG CCC AGT ATA CTT AAG AAA GAA AAG GAG CAA
	 C F N I L S A E A H N R S E N +60
 136	TGC TTT AAT ATT TTG TCT GCA GAA GCA CAT AAT AGA TCT GAA AAC
	 Q E L Q A G C A G E W D N V T +75
 181	CAA GAG CTG CAA GCA GGC TGT GCT GGA GAG TGG GAT AAT GTA ACA
	 C W P S A A V R Q T V S V P C +90
 226	TGT TGG CCC TCT GCT GCT GTA AGA CAG ACT GTC TCT GTA CCC TGT
	 P E F I S L L T G V K D F I Y +105
 271	CCT GAA TTT ATC TCC CTG CTC ACT GGA GTA AAA GAC TTT ATA TAC
	 R N C T I N G W S E A F P R L +120
 316	CGG AAC TGT ACA ATT AAT GGA TGG TCA GAA GCT TTT CCA AGG CTT
	 E T A C G Y K V N D T T T D D +135
 361	GAA ACT GCC TGT GGT TAT AAA GTG AAT GAT ACT ACA ACT GAT GAT
	 K T S Y Y S N L K T M Y T V G +150
 406	AAA ACT TCC TAT TAC TCA AAT TTG AAG ACA ATG TAC ACT GTG GGA
	 Y S T S F L S L S V A V I I L +165
 451	TAC AGC ACA TCC TTT CTG TCA TTA TCT GTT GCA GTC ATA ATT CTA
	 G F F R K L R C T R N Y I H M +180
 496	GGC TTT TTC AGA AAA CTC CGC TGT ACC CGG AAC TAT ATC CAC ATG
	 H L F T S F I L R A L S V L I +195
 541	CAT TTG TTT ACA TCA TTT ATT CTG CGA GCT CTT TCA GTG CTT ATC
	 K D S V L F S S K D I D H C S +210
 586	AAG GAT AGT GTC TTG TTT TCC AGC AAA GAC ATT GAT CAC TGC AGC
	 A Y A S S G C K F V V V F Y Q +225
 631	GCT TAT GCC TCG TCT GGC TGC AAG TTT GTT GTG GTC TTC TAT CAG
	 Y C I M A N Y S W L L V E G L +240
 676	TAC TGC ATT ATG GCT AAC TAC AGC TGG CTA CTA GTG GAA GGA CTC
	 Y L H S L L V I S F F S E R K +255
 721	TAT CTT CAC TCT CTC CTT GTT ATA TCA TTC TTC TCA GAA AGA AAG
	 Y F W W Y I T L G W G L P S V +270
 766	TAC TTT TGG TGG TAT ATC ACA CTT GGA TGG GGT TTA CCT TCC GTT
	 F I I A W S I A R Q L L E D I +285
 811	TTT ATT ATT GCA TGG TCC ATT GCA AGG CAA CTG CTA GAA GAT ATA
	 G C W D I N I N A E I W W I I +300
 856	GGG TGT TGG GAT ATC AAT ATA AAT GCT GAG ATC TGG TGG ATA ATT
	 K G P I I L S I F I N F I L F +315
 901	AAA GGA CCT ATA ATT TTG TCC ATT TTT ATT AAT TTC ATT CTT TTT
	 V S I I R I L I K K L K T P D +330
 946	GTA AGT ATC ATA AGA ATA TTG ATT AAA AAG TTA AAG ACA CCA GAT
	 V R G S N F N Q Y K R L A K S +345
 991	GTG AGA GGA AGC AAC TTT AAT CAG TAC AAG AGA CTT GCA AAA TCC
	 T L L L I P L F G V H Y I T F +360
 1036	ACT TTG TTG TTA ATA CCT TTA TTT GGA GTA CAC TAT ATT ACA TTC
	 T I F P E E V S A V T A E I R +375
 1081	ACA ATT TTT CCT GAG GAA GTT AGT GCT GTC ACA GCA GAA ATT CGA
	 L Y V E L A V G S F Q G F V V +390
 1126	TTA TAT GTT GAA CTT GCT GTT GGA TCT TTC CAG GGG TTT GTC GTT
	 A V L Y C F L N G E V Q S E I +405
 1171	GCT GTT CTC TAC TGT TTC CTT AAT GGA GAG GTT CAA TCT GAA ATC
	 K R K W R R W K L K K H F D V +420
 1216	AAA CGA AAA TGG AGA CGG TGG AAG TTA AAA AAG CAC TTT GAT GTA
	 D F R H P R N S T T S N G T N +435
 1261	GAT TTT CGA CAT CCT CGC AAC TCA ACA ACT AGC AAT GGA ACC AAT
	 G M T L V S L L T R S S P K D +450
 1306	GGC ATG ACC CTG GTT TCC CTG TTA ACC AGG TCT AGT CCA AAA GAT
	 Q R K S F D H S L A E K Y . +463
 1351	CAA AGG AAA TCT TTT GAC CAC AGT CTG GCC GAA AAA TAC TGAaaca
 1397	taaaaaaactgaatttgtgaactataaagtgtaatactgtaaaaaaaaaaaa
TM1
TM2
TM3
TM4
TM5
TM6
TM7
