# Supplementary material for: Origin of Secretin Receptor Precedes the Advent of Tetrapoda: Evidence on the Separated Origins of Secretin and Orexin
Source: PLoS One. 2011 Apr 29;6(4):e19384. doi: 10.1371/journal.pone.0019384 (PMC3084839; doi:10.1371/journal.pone.0019384)
Supplement: Figure S3 — Percent amino acid homology of vertebrate secretin receptor (A) ligand-binding domain and (B) entire sequence. (PPTX) [file pone.0019384.s003.pptx]

## Slide 1
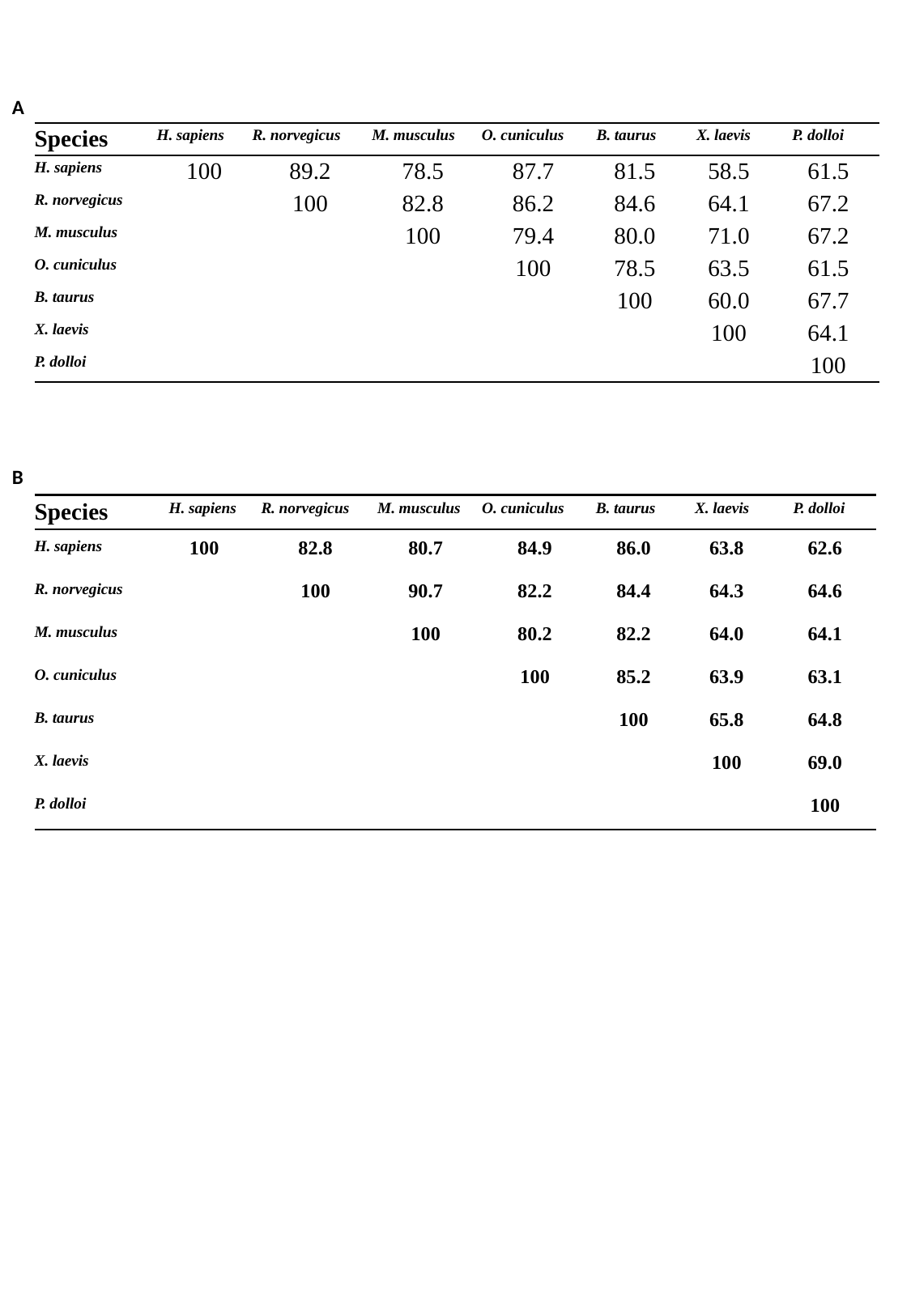

A
| Species | H. sapiens | | R. norvegicus | M. musculus | O. cuniculus | B. taurus | X. laevis | P. dolloi |
| --- | --- | --- | --- | --- | --- | --- | --- | --- |
| H. sapiens | | 100 | 89.2 | 78.5 | 87.7 | 81.5 | 58.5 | 61.5 |
| R. norvegicus | | | 100 | 82.8 | 86.2 | 84.6 | 64.1 | 67.2 |
| M. musculus | | | | 100 | 79.4 | 80.0 | 71.0 | 67.2 |
| O. cuniculus | | | | | 100 | 78.5 | 63.5 | 61.5 |
| B. taurus | | | | | | 100 | 60.0 | 67.7 |
| X. laevis | | | | | | | 100 | 64.1 |
| P. dolloi | | | | | | | | 100 |
B
| Species | | H. sapiens | R. norvegicus | M. musculus | O. cuniculus | | B. taurus | X. laevis | P. dolloi |
| --- | --- | --- | --- | --- | --- | --- | --- | --- | --- |
| H. sapiens | 100 | | 82.8 | 80.7 | | 84.9 | 86.0 | 63.8 | 62.6 |
| R. norvegicus | | | 100 | 90.7 | | 82.2 | 84.4 | 64.3 | 64.6 |
| M. musculus | | | | 100 | | 80.2 | 82.2 | 64.0 | 64.1 |
| O. cuniculus | | | | | | 100 | 85.2 | 63.9 | 63.1 |
| B. taurus | | | | | | | 100 | 65.8 | 64.8 |
| X. laevis | | | | | | | | 100 | 69.0 |
| P. dolloi | | | | | | | | | 100 |
