# Supplementary material for: Origin of Secretin Receptor Precedes the Advent of Tetrapoda: Evidence on the Separated Origins of Secretin and Orexin
Source: PLoS One. 2011 Apr 29;6(4):e19384. doi: 10.1371/journal.pone.0019384 (PMC3084839; doi:10.1371/journal.pone.0019384)
Supplement: Figure S4 — Alignment of cloned secretin receptor amino acid sequences. Putative transmembrane domains are overlined and labeled. # and * indicate potential sites for N-linked glycosylation and conserved cysteine residues, respectively. Predicted ligand-binding domains are indicated in bold characters. Structural features are boxed with dotted lines. Gaps (represented by - ) were introduced to maximize sequence homology. (PPTX) [file pone.0019384.s004.pptx]

## Slide 1
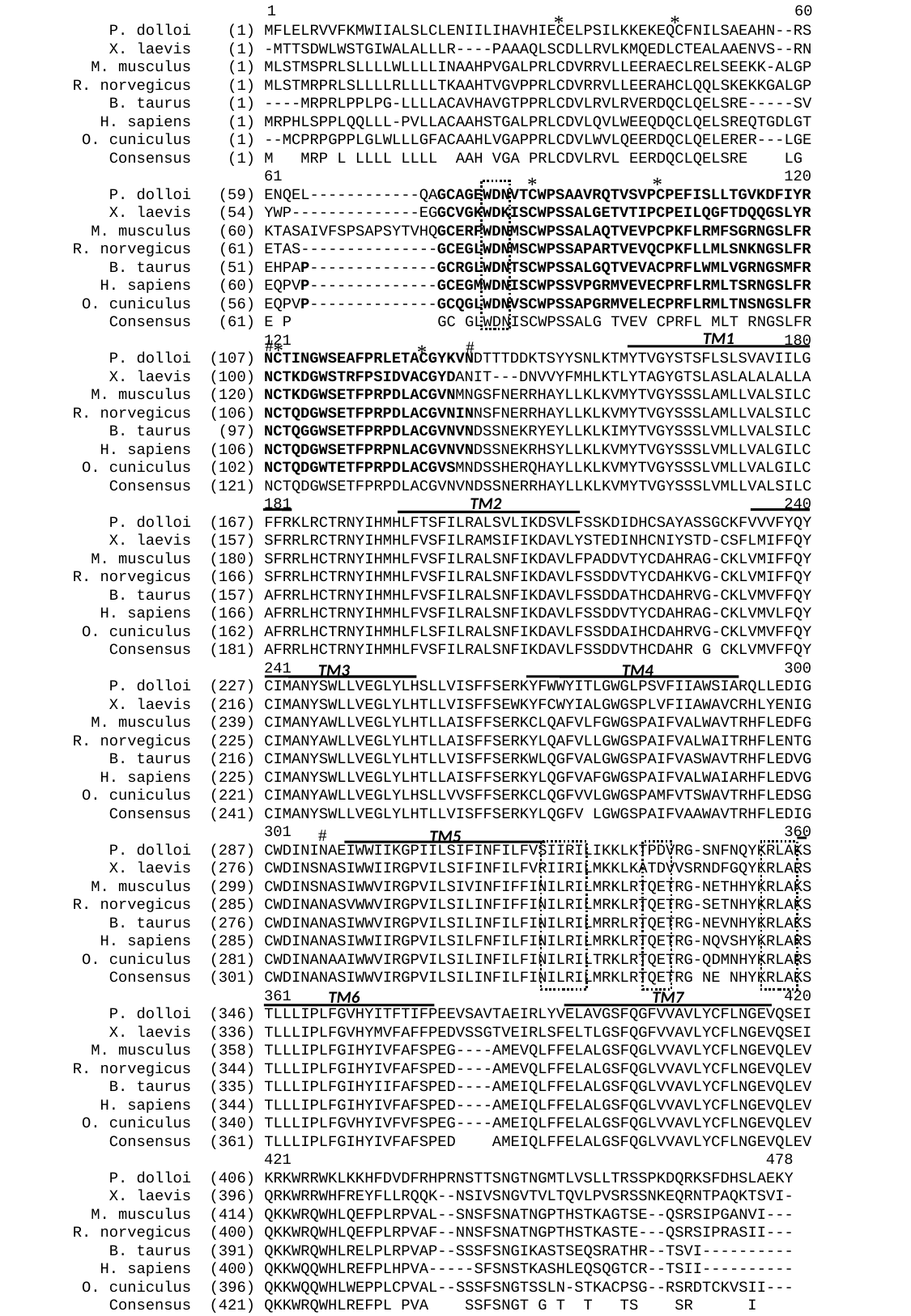

1 60
 P. dolloi (1) MFLELRVVFKMWIIALSLCLENIILIHAVHIECELPSILKKEKEQCFNILSAEAHN--RS
 X. laevis (1) -MTTSDWLWSTGIWALALLLR----PAAAQLSCDLLRVLKMQEDLCTEALAAENVS--RN
 M. musculus (1) MLSTMSPRLSLLLLWLLLLINAAHPVGALPRLCDVRRVLLEERAECLRELSEEKK-ALGP
R. norvegicus (1) MLSTMRPRLSLLLLRLLLLTKAAHTVGVPPRLCDVRRVLLEERAHCLQQLSKEKKGALGP
 B. taurus (1) ----MRPRLPPLPG-LLLLACAVHAVGTPPRLCDVLRVLRVERDQCLQELSRE-----SV
 H. sapiens (1) MRPHLSPPLQQLLL-PVLLACAAHSTGALPRLCDVLQVLWEEQDQCLQELSREQTGDLGT
 O. cuniculus (1) --MCPRPGPPLGLWLLLGFACAAHLVGAPPRLCDVLWVLQEERDQCLQELERER---LGE
 Consensus (1) M MRP L LLLL LLLL AAH VGA PRLCDVLRVL EERDQCLQELSRE LG
 61 120
 P. dolloi (59) ENQEL------------QAGCAGEWDNVTCWPSAAVRQTVSVPCPEFISLLTGVKDFIYR
 X. laevis (54) YWP--------------EGGCVGKWDKISCWPSSALGETVTIPCPEILQGFTDQQGSLYR
 M. musculus (60) KTASAIVFSPSAPSYTVHQGCERFWDNMSCWPSSALAQTVEVPCPKFLRMFSGRNGSLFR
R. norvegicus (61) ETAS---------------GCEGLWDNMSCWPSSAPARTVEVQCPKFLLMLSNKNGSLFR
 B. taurus (51) EHPAP--------------GCRGLWDNTSCWPSSALGQTVEVACPRFLWMLVGRNGSMFR
 H. sapiens (60) EQPVP--------------GCEGMWDNISCWPSSVPGRMVEVECPRFLRMLTSRNGSLFR
 O. cuniculus (56) EQPVP--------------GCQGLWDNVSCWPSSAPGRMVELECPRFLRMLTNSNGSLFR
 Consensus (61) E P GC GLWDNISCWPSSALG TVEV CPRFL MLT RNGSLFR
 121 180
 P. dolloi (107) NCTINGWSEAFPRLETACGYKVNDTTTDDKTSYYSNLKTMYTVGYSTSFLSLSVAVIILG
 X. laevis (100) NCTKDGWSTRFPSIDVACGYDANIT---DNVVYFMHLKTLYTAGYGTSLASLALALALLA
 M. musculus (120) NCTKDGWSETFPRPDLACGVNMNGSFNERRHAYLLKLKVMYTVGYSSSLAMLLVALSILC
R. norvegicus (106) NCTQDGWSETFPRPDLACGVNINNSFNERRHAYLLKLKVMYTVGYSSSLAMLLVALSILC
 B. taurus (97) NCTQGGWSETFPRPDLACGVNVNDSSNEKRYEYLLKLKIMYTVGYSSSLVMLLVALSILC
 H. sapiens (106) NCTQDGWSETFPRPNLACGVNVNDSSNEKRHSYLLKLKVMYTVGYSSSLVMLLVALGILC
 O. cuniculus (102) NCTQDGWTETFPRPDLACGVSMNDSSHERQHAYLLKLKVMYTVGYSSSLVMLLVALGILC
 Consensus (121) NCTQDGWSETFPRPDLACGVNVNDSSNERRHAYLLKLKVMYTVGYSSSLVMLLVALSILC
 181 240
 P. dolloi (167) FFRKLRCTRNYIHMHLFTSFILRALSVLIKDSVLFSSKDIDHCSAYASSGCKFVVVFYQY
 X. laevis (157) SFRRLRCTRNYIHMHLFVSFILRAMSIFIKDAVLYSTEDINHCNIYSTD-CSFLMIFFQY
 M. musculus (180) SFRRLHCTRNYIHMHLFVSFILRALSNFIKDAVLFPADDVTYCDAHRAG-CKLVMIFFQY
R. norvegicus (166) SFRRLHCTRNYIHMHLFVSFILRALSNFIKDAVLFSSDDVTYCDAHKVG-CKLVMIFFQY
 B. taurus (157) AFRRLHCTRNYIHMHLFVSFILRALSNFIKDAVLFSSDDATHCDAHRVG-CKLVMVFFQY
 H. sapiens (166) AFRRLHCTRNYIHMHLFVSFILRALSNFIKDAVLFSSDDVTYCDAHRAG-CKLVMVLFQY
 O. cuniculus (162) AFRRLHCTRNYIHMHLFLSFILRALSNFIKDAVLFSSDDAIHCDAHRVG-CKLVMVFFQY
 Consensus (181) AFRRLHCTRNYIHMHLFVSFILRALSNFIKDAVLFSSDDVTHCDAHR G CKLVMVFFQY
 241 300
 P. dolloi (227) CIMANYSWLLVEGLYLHSLLVISFFSERKYFWWYITLGWGLPSVFIIAWSIARQLLEDIG
 X. laevis (216) CIMANYSWLLVEGLYLHTLLVISFFSEWKYFCWYIALGWGSPLVFIIAWAVCRHLYENIG
 M. musculus (239) CIMANYAWLLVEGLYLHTLLAISFFSERKCLQAFVLFGWGSPAIFVALWAVTRHFLEDFG
R. norvegicus (225) CIMANYAWLLVEGLYLHTLLAISFFSERKYLQAFVLLGWGSPAIFVALWAITRHFLENTG
 B. taurus (216) CIMANYSWLLVEGLYLHTLLVISFFSERKWLQGFVALGWGSPAIFVASWAVTRHFLEDVG
 H. sapiens (225) CIMANYSWLLVEGLYLHTLLAISFFSERKYLQGFVAFGWGSPAIFVALWAIARHFLEDVG
 O. cuniculus (221) CIMANYAWLLVEGLYLHSLLVVSFFSERKCLQGFVVLGWGSPAMFVTSWAVTRHFLEDSG
 Consensus (241) CIMANYSWLLVEGLYLHTLLVISFFSERKYLQGFV LGWGSPAIFVAAWAVTRHFLEDIG
 301 360
 P. dolloi (287) CWDININAEIWWIIKGPIILSIFINFILFVSIIRILIKKLKTPDVRG-SNFNQYKRLAKS
 X. laevis (276) CWDINSNASIWWIIRGPVILSIFINFILFVRIIRILMKKLKATDVVSRNDFGQYKRLARS
 M. musculus (299) CWDINSNASIWWVIRGPVILSIVINFIFFINILRILMRKLRTQETRG-NETHHYKRLAKS
R. norvegicus (285) CWDINANASVWWVIRGPVILSILINFIFFINILRILMRKLRTQETRG-SETNHYKRLAKS
 B. taurus (276) CWDINANASIWWVIRGPVILSILINFILFINILRILMRRLRTQETRG-NEVNHYKRLAKS
 H. sapiens (285) CWDINANASIWWIIRGPVILSILFNFILFINILRILMRKLRTQETRG-NQVSHYKRLARS
 O. cuniculus (281) CWDINANAAIWWVIRGPVILSILINFILFINILRILTRKLRTQETRG-QDMNHYKRLARS
 Consensus (301) CWDINANASIWWVIRGPVILSILINFILFINILRILMRKLRTQETRG NE NHYKRLAKS
 361 420
 P. dolloi (346) TLLLIPLFGVHYITFTIFPEEVSAVTAEIRLYVELAVGSFQGFVVAVLYCFLNGEVQSEI
 X. laevis (336) TLLLIPLFGVHYMVFAFFPEDVSSGTVEIRLSFELTLGSFQGFVVAVLYCFLNGEVQSEI
 M. musculus (358) TLLLIPLFGIHYIVFAFSPEG----AMEVQLFFELALGSFQGLVVAVLYCFLNGEVQLEV
R. norvegicus (344) TLLLIPLFGIHYIVFAFSPED----AMEVQLFFELALGSFQGLVVAVLYCFLNGEVQLEV
 B. taurus (335) TLLLIPLFGIHYIIFAFSPED----AMEIQLFFELALGSFQGLVVAVLYCFLNGEVQLEV
 H. sapiens (344) TLLLIPLFGIHYIVFAFSPED----AMEIQLFFELALGSFQGLVVAVLYCFLNGEVQLEV
 O. cuniculus (340) TLLLIPLFGVHYIVFVFSPEG----AMEIQLFFELALGSFQGLVVAVLYCFLNGEVQLEV
 Consensus (361) TLLLIPLFGIHYIVFAFSPED AMEIQLFFELALGSFQGLVVAVLYCFLNGEVQLEV
 421 478
 P. dolloi (406) KRKWRRWKLKKHFDVDFRHPRNSTTSNGTNGMTLVSLLTRSSPKDQRKSFDHSLAEKY
 X. laevis (396) QRKWRRWHFREYFLLRQQK--NSIVSNGVTVLTQVLPVSRSSNKEQRNTPAQKTSVI-
 M. musculus (414) QKKWRQWHLQEFPLRPVAL--SNSFSNATNGPTHSTKAGTSE--QSRSIPGANVI---
R. norvegicus (400) QKKWRQWHLQEFPLRPVAF--NNSFSNATNGPTHSTKASTE---QSRSIPRASII---
 B. taurus (391) QKKWRQWHLRELPLRPVAP--SSSFSNGIKASTSEQSRATHR--TSVI----------
 H. sapiens (400) QKKWQQWHLREFPLHPVA-----SFSNSTKASHLEQSQGTCR--TSII----------
 O. cuniculus (396) QKKWQQWHLWEPPLCPVAL--SSSFSNGTSSLN-STKACPSG--RSRDTCKVSII---
 Consensus (421) QKKWRQWHLREFPL PVA SSFSNGT G T T TS SR I
*
*
*
*
TM1
#
#
*
*
TM2
TM3
TM4
#
TM5
TM6
TM7
