# Supplementary figures and images for: Origin of Secretin Receptor Precedes the Advent of Tetrapoda: Evidence on the Separated Origins of Secretin and Orexin
Source: PLoS One. 2011 Apr 29;6(4):e19384. doi: 10.1371/journal.pone.0019384 (PMC3084839; doi:10.1371/journal.pone.0019384)

## Slide 1
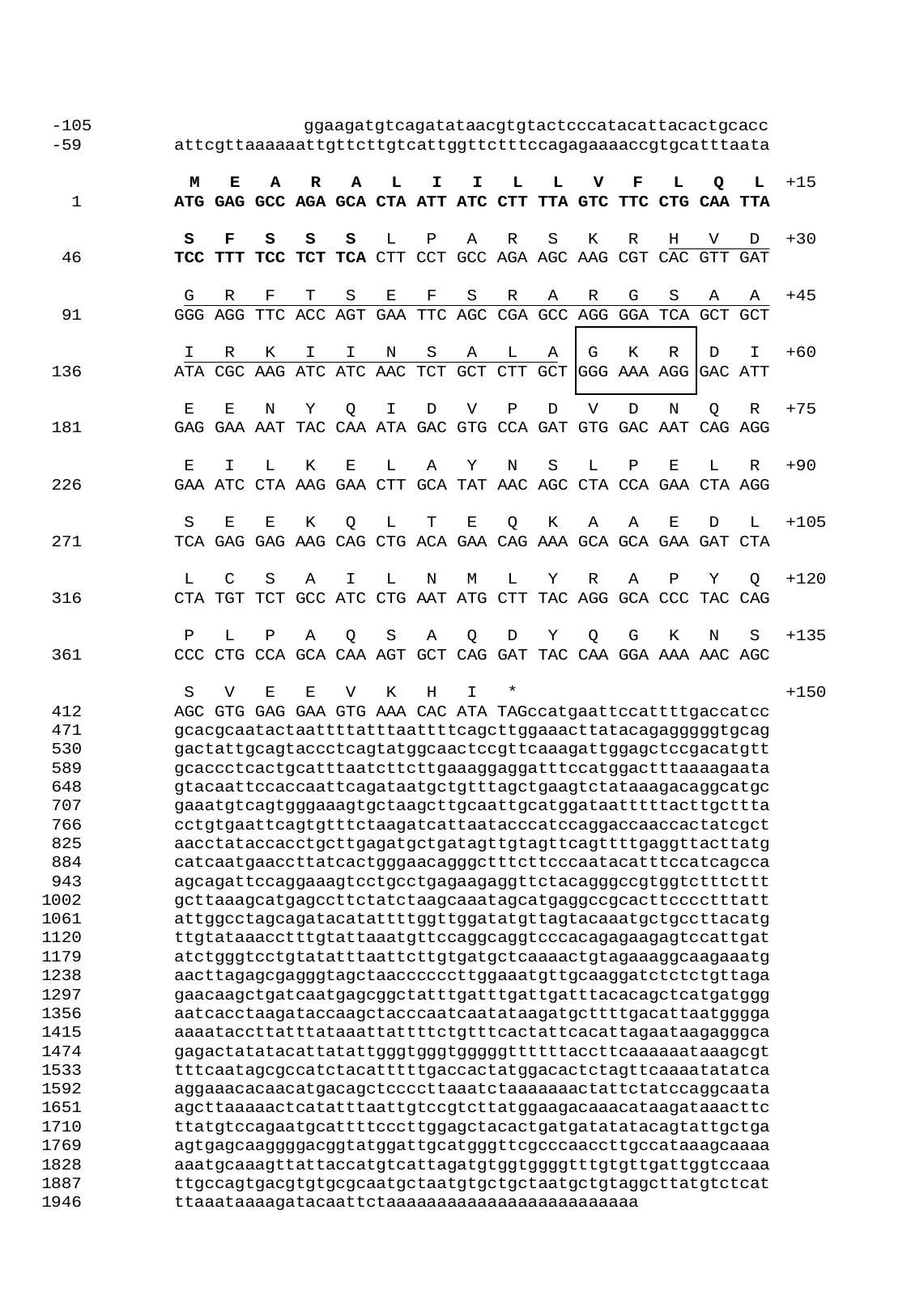

Supplement: Figure S5 — Nucleotide (GenBank accession no. HQ236553) and deduced amino acid sequence of the X. laevis secretin (xSCT) cDNA. The full-length xSCT is 2141 bp in length. Nucleotides (lower line) and amino acids (upper line) are numbered from the initiation methionine residue. The signal peptide (20 amino acids) is indicated in bold characters. The mature peptide (28 amino acids) is underlined with solid line and the potential cleavage/amidation site (GKR) is boxed. (PPTX) [file pone.0019384.s005.pptx]

## Slide 1
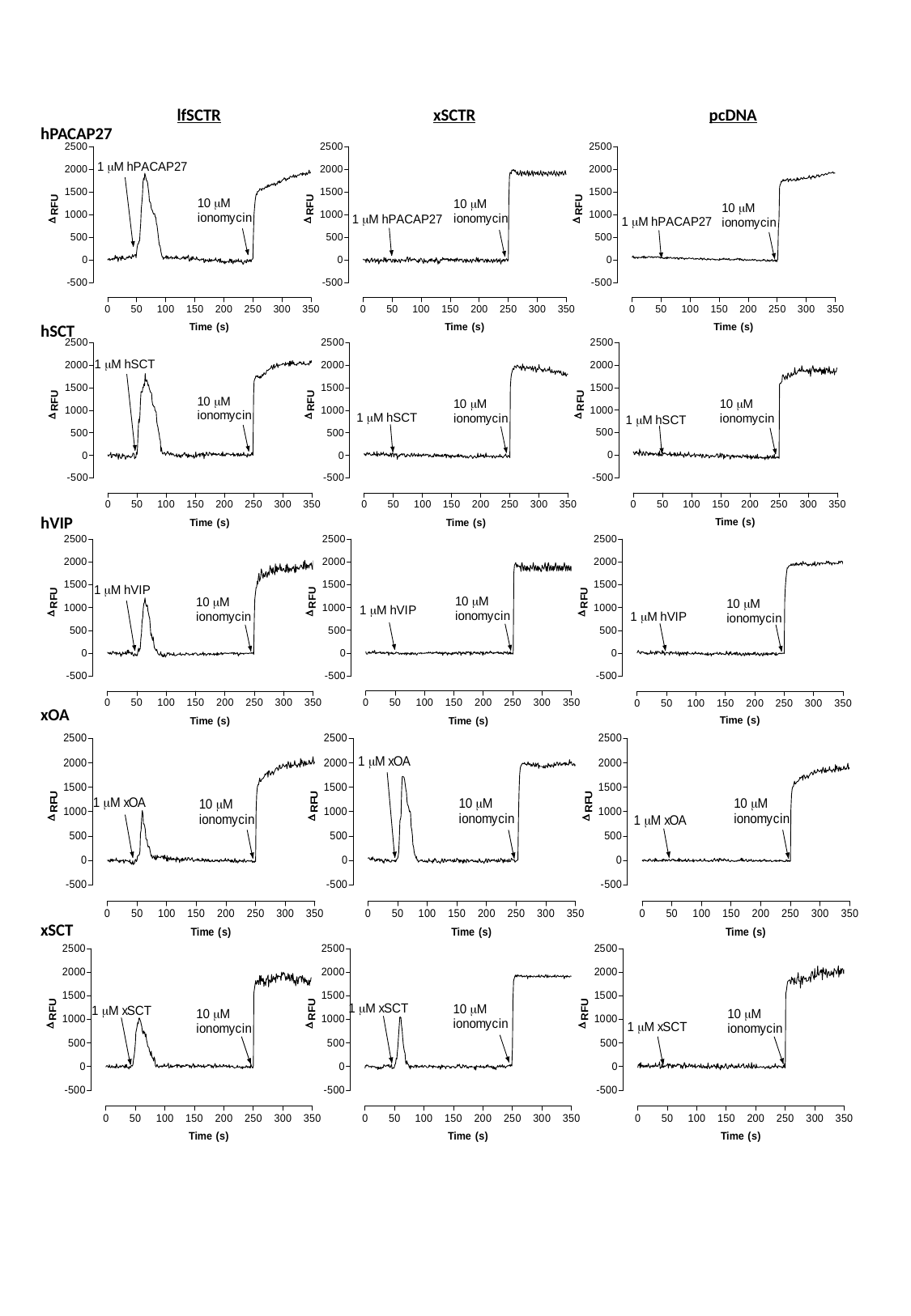

lfSCTR
xSCTR
pcDNA
hPACAP27
hSCT
hVIP
xOA
xSCT

Supplement: Figure S8 — Representative traces of hPACAP27, hSCT, hVIP, xOA, and xSCT on intracellular calcium mobilization in lfSCTR, xSCTR and null pcDNA 3.1-transfected CHO cells. Peak magnitude of traces is proportional to the order of potency of the ligands tested. Traces were obtained from at least 10 calcium assays with respective control shown on the right panel. Ionomycin (10 µM) was added at the end of each experiment to test the vitality of the cells. (PPTX) [file pone.0019384.s008.pptx]

## Slide 1
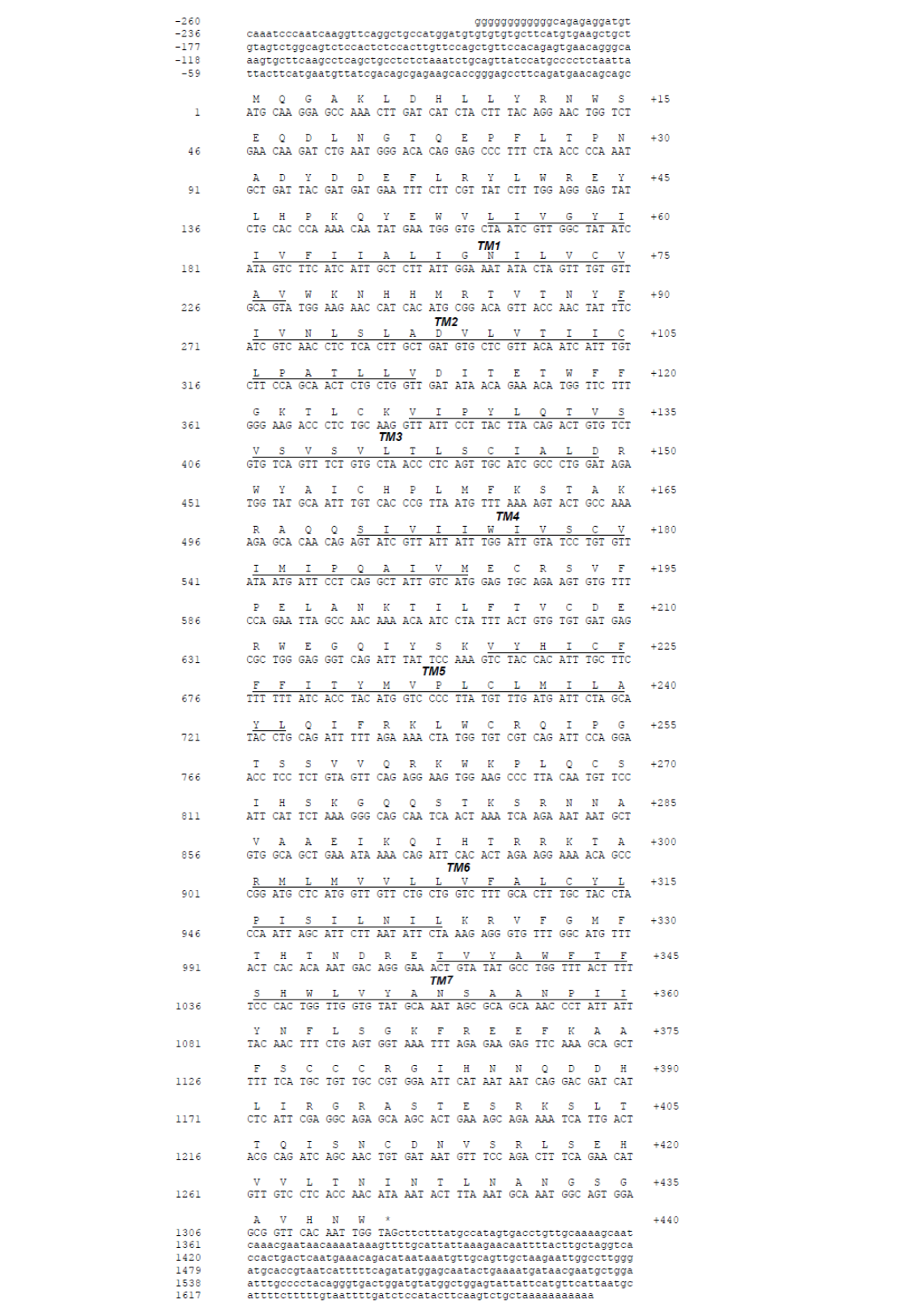

Supplement: Figure S9 — Full-length nucleotide (GenBank accession no. HQ242647) and deduced amino acid sequence of X. laevis orexin type-2 receptor (xOX2R). Numbers on the left and right indicate the position of the first nucleotide and the last amino acid of each line from the start codon, respectively. The ORF sequence is presented in upper cases whereas the 3′ and 5′ UTR sequences are presented in lower cases. The amino acid sequences corresponding to the seven putative transmembrane domains (TM1-7) are labeled and underlined. The stop codon is marked by an asterisk sign (*). (PPTX) [file pone.0019384.s009.pptx]

## Slide 1
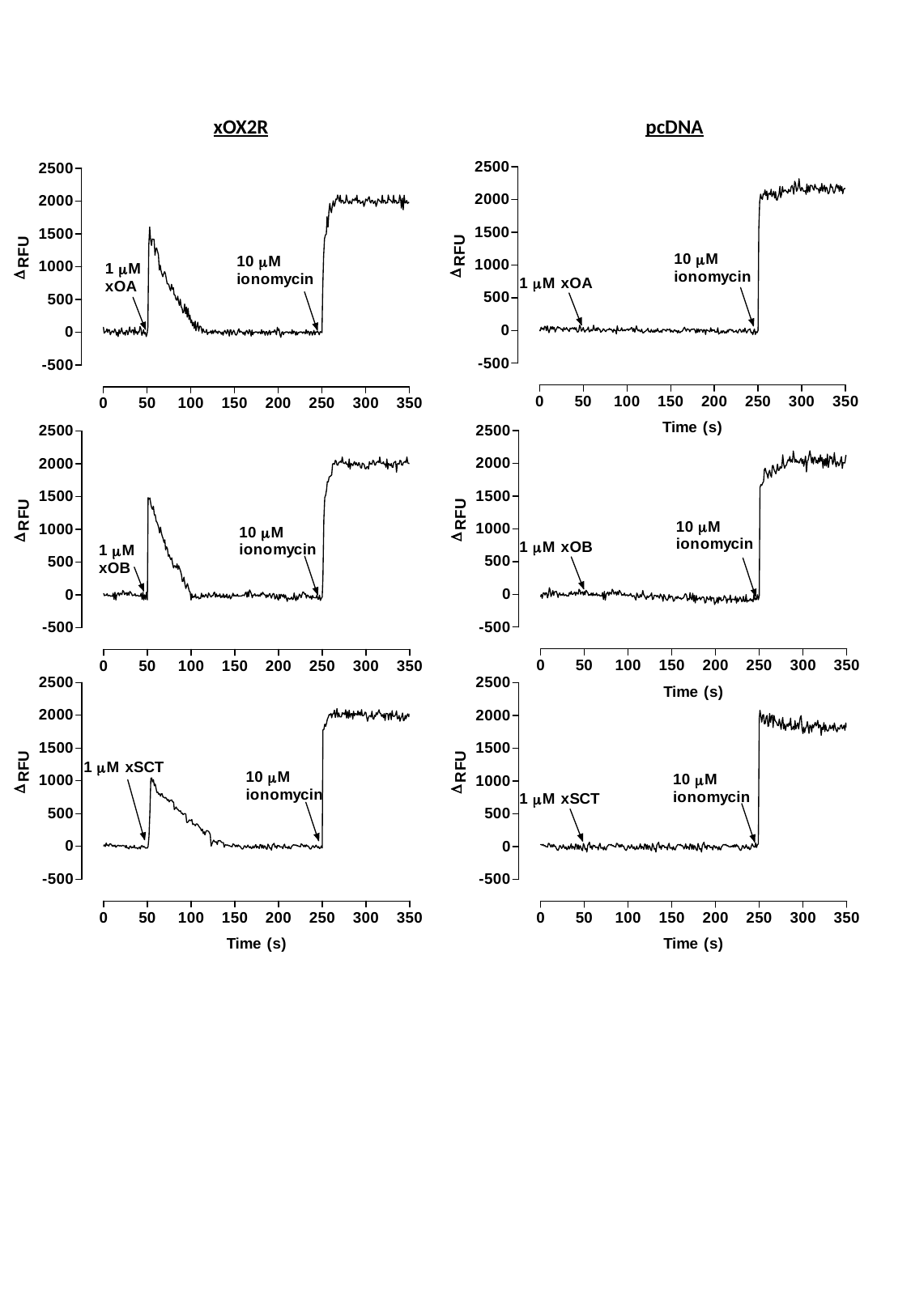

xOX2R
pcDNA

Supplement: Figure S10 — Representative traces of xSCT, xOA, and xOB on intracellular calcium mobilization in xOX2R- and null pcDNA 3.1-transfected CHO cells. Peak magnitude of traces is proportional to the order of potency of the ligands tested. Traces were obtained from at least 10 calcium assays with respective control shown on the right panel. Ionomycin (10 µM) was added at the end of each experiment to test the vitality of the cells. (PPTX) [file pone.0019384.s010.pptx]
