# Supplementary material for: Origin of Secretin Receptor Precedes the Advent of Tetrapoda: Evidence on the Separated Origins of Secretin and Orexin
Source: PLoS One. 2011 Apr 29;6(4):e19384. doi: 10.1371/journal.pone.0019384 (PMC3084839; doi:10.1371/journal.pone.0019384)
Supplement: Figure S6 — Nucleotide (GenBank accession no. HQ236554) and deduced amino acid sequence of the R. rugulosa secretin (Rana SCT) cDNA. The full-length Rana SCT is 763 bp in length. Nucleotides (lower line) and amino acids (upper line) are numbered from the initiation methionine residue. The signal peptide (22 amino acids) is indicated in bold characters. The mature peptide (28 amino acids) is underlined with solid line and the potential cleavage/amidation site (GKR) is boxed. (PPTX) [file pone.0019384.s006.pptx]

## Slide 1
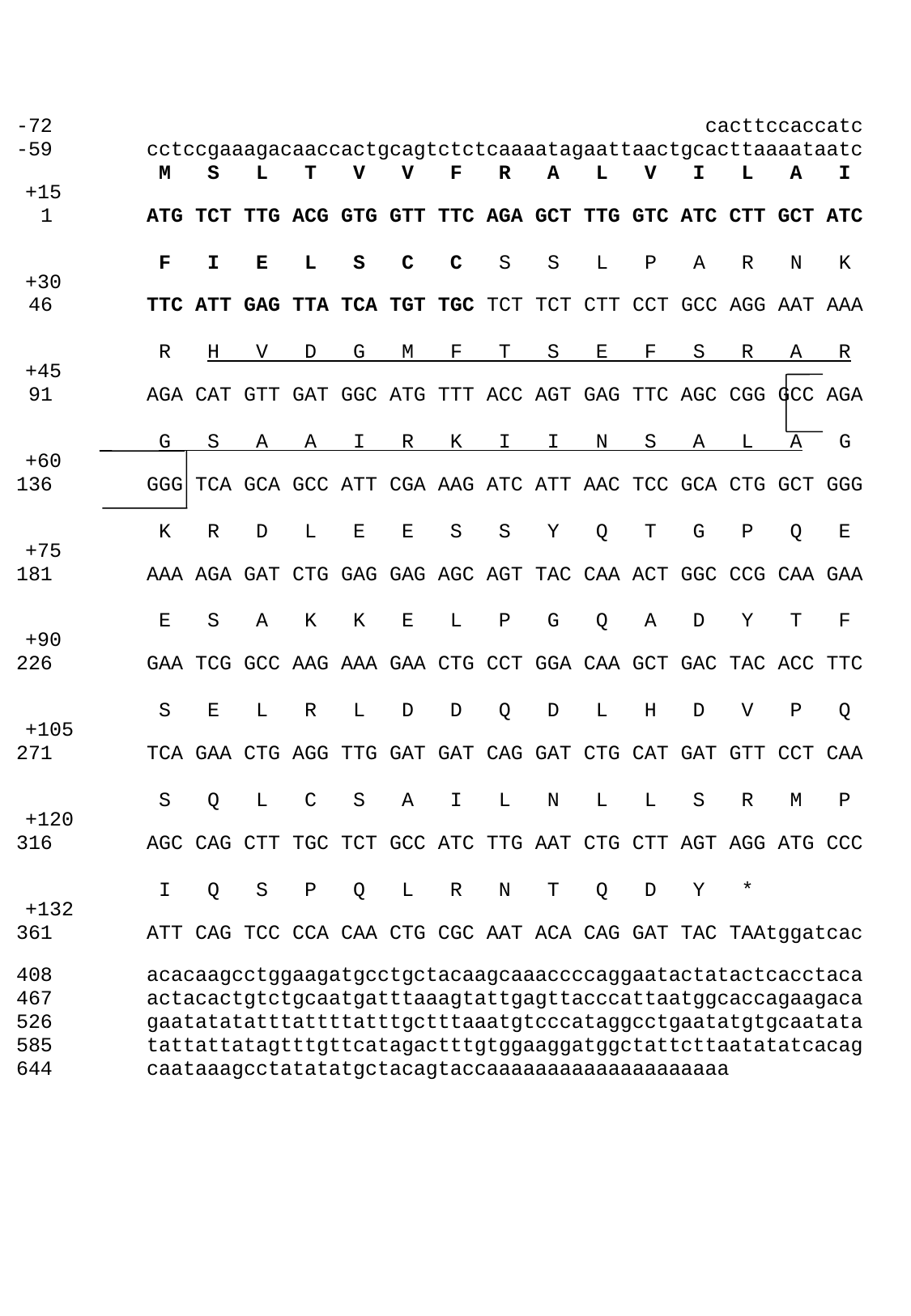

-72			 cacttccaccatc
 -59 	cctccgaaagacaaccactgcagtctctcaaaatagaattaactgcacttaaaataatc
		 M S L T V V F R A L V I L A I	 +15
 1	ATG TCT TTG ACG GTG GTT TTC AGA GCT TTG GTC ATC CTT GCT ATC
		 F I E L S C C S S L P A R N K	 +30
 46	TTC ATT GAG TTA TCA TGT TGC TCT TCT CTT CCT GCC AGG AAT AAA
		 R H V D G M F T S E F S R A R	 +45
 91	AGA CAT GTT GAT GGC ATG TTT ACC AGT GAG TTC AGC CGG GCC AGA
		 G S A A I R K I I N S A L A G +60
 136	GGG TCA GCA GCC ATT CGA AAG ATC ATT AAC TCC GCA CTG GCT GGG
		 K R D L E E S S Y Q T G P Q E +75
 181	AAA AGA GAT CTG GAG GAG AGC AGT TAC CAA ACT GGC CCG CAA GAA
		 E S A K K E L P G Q A D Y T F +90
 226	GAA TCG GCC AAG AAA GAA CTG CCT GGA CAA GCT GAC TAC ACC TTC
		 S E L R L D D Q D L H D V P Q +105
 271	TCA GAA CTG AGG TTG GAT GAT CAG GAT CTG CAT GAT GTT CCT CAA
		 S Q L C S A I L N L L S R M P +120
 316	AGC CAG CTT TGC TCT GCC ATC TTG AAT CTG CTT AGT AGG ATG CCC
		 I Q S P Q L R N T Q D Y * +132
 361	ATT CAG TCC CCA CAA CTG CGC AAT ACA CAG GAT TAC TAAtggatcac
 408	acacaagcctggaagatgcctgctacaagcaaaccccaggaatactatactcacctaca
 467	actacactgtctgcaatgatttaaagtattgagttacccattaatggcaccagaagaca
 526	gaatatatatttattttatttgctttaaatgtcccataggcctgaatatgtgcaatata
 585	tattattatagtttgttcatagactttgtggaaggatggctattcttaatatatcacag
 644	caataaagcctatatatgctacagtaccaaaaaaaaaaaaaaaaaaaa
