# Supplementary material for: Origin of Secretin Receptor Precedes the Advent of Tetrapoda: Evidence on the Separated Origins of Secretin and Orexin
Source: PLoS One. 2011 Apr 29;6(4):e19384. doi: 10.1371/journal.pone.0019384 (PMC3084839; doi:10.1371/journal.pone.0019384)
Supplement: Figure S7 — Sequence analyses of secretin mature peptides. (A) Alignment of secretin mature peptide sequences. Identical residues are indicated in bold characters. Accession numbers are: H. sapiens, AAG31443; R. norvegicus, AAA42128; M. musculus, CAA51982; B. taurus, P63296; C. familiaris, P09910; O. cuniculus, P32647; S. scrofa, AAA31121; G. gallus, NP_001020004. (B) Percent amino acid homology of the aligned secretin mature peptides. (PPTX) [file pone.0019384.s007.pptx]

## Slide 1
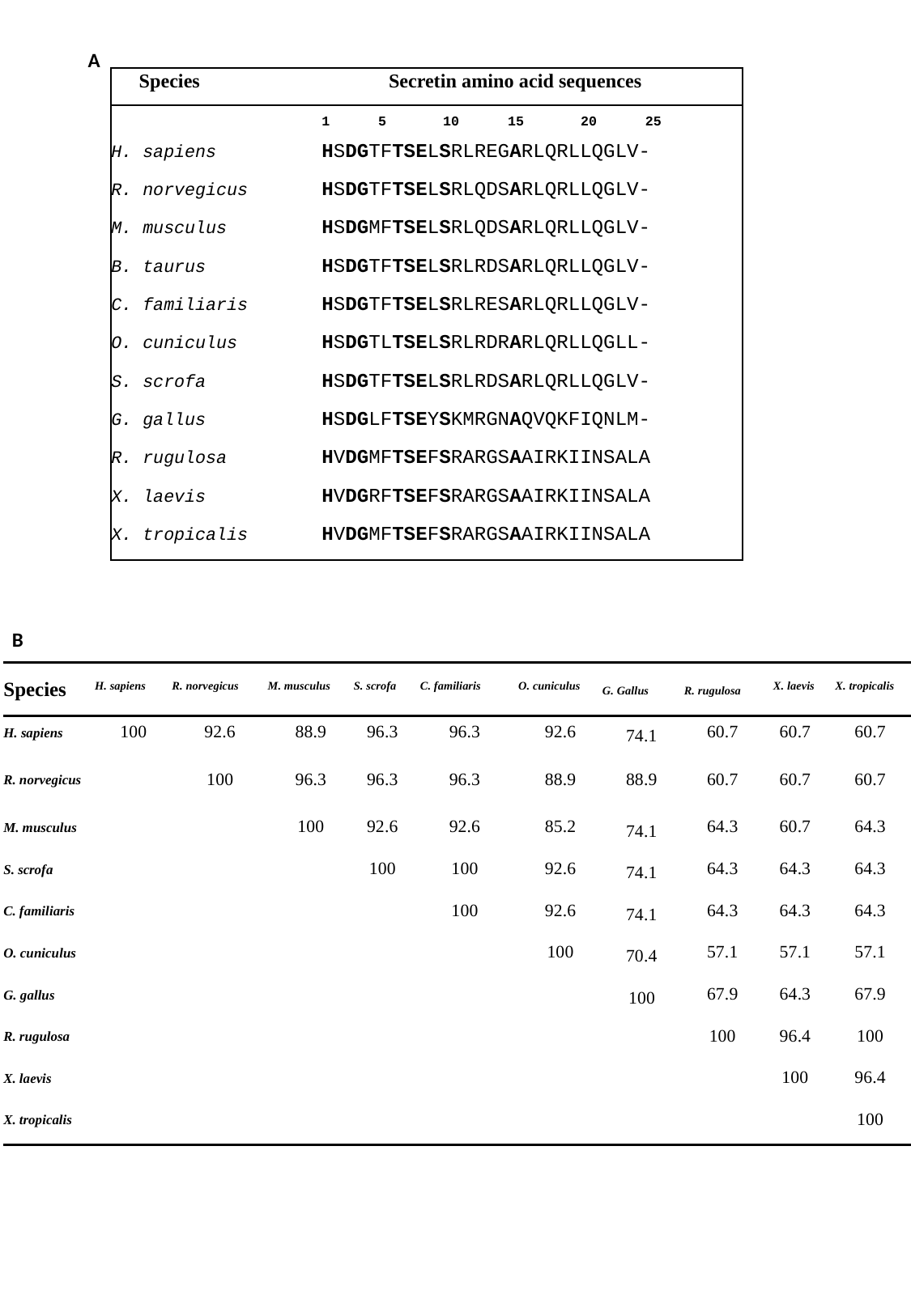

A
| Species | Secretin amino acid sequences | |
| --- | --- | --- |
| | | 1 5 10 15 20 25 |
| H. sapiens | | HSDGTFTSELSRLREGARLQRLLQGLV- |
| R. norvegicus | | HSDGTFTSELSRLQDSARLQRLLQGLV- |
| M. musculus | | HSDGMFTSELSRLQDSARLQRLLQGLV- |
| B. taurus | | HSDGTFTSELSRLRDSARLQRLLQGLV- |
| C. familiaris | | HSDGTFTSELSRLRESARLQRLLQGLV- |
| O. cuniculus | | HSDGTLTSELSRLRDRARLQRLLQGLL- |
| S. scrofa | | HSDGTFTSELSRLRDSARLQRLLQGLV- |
| G. gallus | | HSDGLFTSEYSKMRGNAQVQKFIQNLM- |
| R. rugulosa | | HVDGMFTSEFSRARGSAAIRKIINSALA |
| X. laevis | | HVDGRFTSEFSRARGSAAIRKIINSALA |
| X. tropicalis | | HVDGMFTSEFSRARGSAAIRKIINSALA |
B
| Species | H. sapiens | R. norvegicus | M. musculus | S. scrofa | C. familiaris | O. cuniculus | G. Gallus | R. rugulosa | X. laevis | X. tropicalis |
| --- | --- | --- | --- | --- | --- | --- | --- | --- | --- | --- |
| H. sapiens | 100 | 92.6 | 88.9 | 96.3 | 96.3 | 92.6 | 74.1 | 60.7 | 60.7 | 60.7 |
| R. norvegicus | | 100 | 96.3 | 96.3 | 96.3 | 88.9 | 88.9 | 60.7 | 60.7 | 60.7 |
| M. musculus | | | 100 | 92.6 | 92.6 | 85.2 | 74.1 | 64.3 | 60.7 | 64.3 |
| S. scrofa | | | | 100 | 100 | 92.6 | 74.1 | 64.3 | 64.3 | 64.3 |
| C. familiaris | | | | | 100 | 92.6 | 74.1 | 64.3 | 64.3 | 64.3 |
| O. cuniculus | | | | | | 100 | 70.4 | 57.1 | 57.1 | 57.1 |
| G. gallus | | | | | | | 100 | 67.9 | 64.3 | 67.9 |
| R. rugulosa | | | | | | | | 100 | 96.4 | 100 |
| X. laevis | | | | | | | | | 100 | 96.4 |
| X. tropicalis | | | | | | | | | | 100 |
