# Supplementary material for: Origin of Secretin Receptor Precedes the Advent of Tetrapoda: Evidence on the Separated Origins of Secretin and Orexin
Source: PLoS One. 2011 Apr 29;6(4):e19384. doi: 10.1371/journal.pone.0019384 (PMC3084839; doi:10.1371/journal.pone.0019384)
Supplement: Table S1 — List of primers used in PCR and real-time PCR amplifications. (PPTX) [file pone.0019384.s011.pptx]

## Slide 1
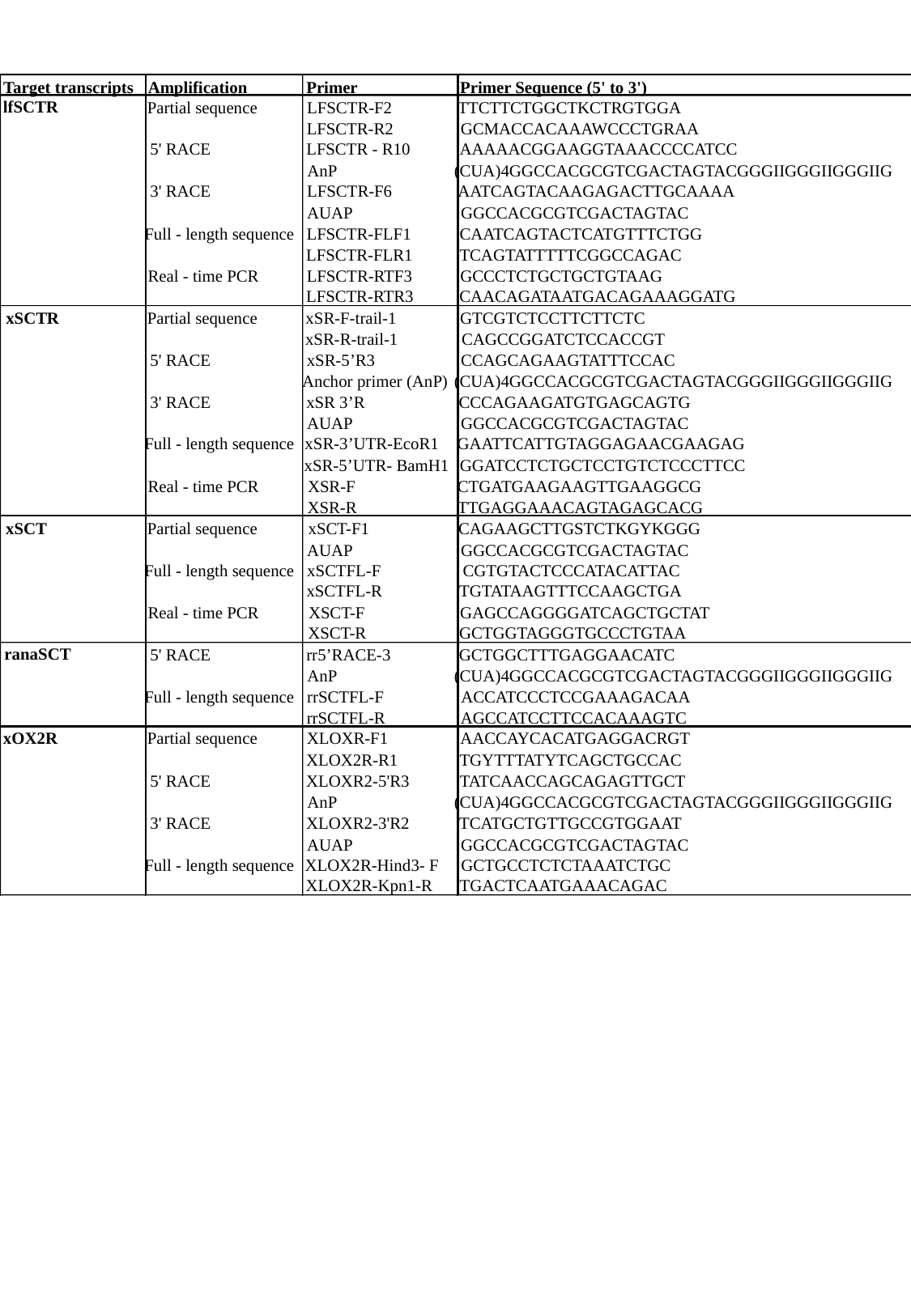

Target transcripts
Amplification
Primer
Primer Sequence (5' to 3')
lfSCTR
Partial sequence
LFSCTR-F2
TTCTTCTGGCTKCTRGTGGA
LFSCTR-R2
GCMACCACAAAWCCCTGRAA
5' RACE
LFSCTR - R10
AAAAACGGAAGGTAAACCCCATCC
AnP
(CUA)4GGCCACGCGTCGACTAGTACGGGIIGGGIIGGGIIG
3' RACE
LFSCTR-F6
AATCAGTACAAGAGACTTGCAAAA
AUAP
GGCCACGCGTCGACTAGTAC
Full - length sequence
LFSCTR-FLF1
CAATCAGTACTCATGTTTCTGG
LFSCTR-FLR1
TCAGTATTTTTCGGCCAGAC
Real - time PCR
LFSCTR-RTF3
GCCCTCTGCTGCTGTAAG
LFSCTR-RTR3
CAACAGATAATGACAGAAAGGATG
xSCTR
Partial sequence
xSR-F-trail-1
GTCGTCTCCTTCTTCTC
xSR-R-trail-1
CAGCCGGATCTCCACCGT
5' RACE
xSR-5’R3
CCAGCAGAAGTATTTCCAC
Anchor primer (AnP)
(CUA)4GGCCACGCGTCGACTAGTACGGGIIGGGIIGGGIIG
3' RACE
xSR 3’R
CCCAGAAGATGTGAGCAGTG
AUAP
GGCCACGCGTCGACTAGTAC
Full - length sequence
xSR-3’UTR-EcoR1
GAATTCATTGTAGGAGAACGAAGAG
xSR-5’UTR- BamH1
GGATCCTCTGCTCCTGTCTCCCTTCC
Real - time PCR
XSR-F
CTGATGAAGAAGTTGAAGGCG
XSR-R
TTGAGGAAACAGTAGAGCACG
xSCT
Partial sequence
xSCT-F1
CAGAAGCTTGSTCTKGYKGGG
AUAP
GGCCACGCGTCGACTAGTAC
Full - length sequence
xSCTFL-F
CGTGTACTCCCATACATTAC
xSCTFL-R
TGTATAAGTTTCCAAGCTGA
Real - time PCR
XSCT-F
GAGCCAGGGGATCAGCTGCTAT
XSCT-R
GCTGGTAGGGTGCCCTGTAA
ranaSCT
5' RACE
rr5’RACE-3
GCTGGCTTTGAGGAACATC
AnP
(CUA)4GGCCACGCGTCGACTAGTACGGGIIGGGIIGGGIIG
Full - length sequence
rrSCTFL-F
ACCATCCCTCCGAAAGACAA
rrSCTFL-R
AGCCATCCTTCCACAAAGTC
xOX2R
Partial sequence
XLOXR-F1
AACCAYCACATGAGGACRGT
XLOX2R-R1
TGYTTTATYTCAGCTGCCAC
5' RACE
XLOXR2-5'R3
TATCAACCAGCAGAGTTGCT
AnP
(CUA)4GGCCACGCGTCGACTAGTACGGGIIGGGIIGGGIIG
3' RACE
XLOXR2-3'R2
TCATGCTGTTGCCGTGGAAT
AUAP
GGCCACGCGTCGACTAGTAC
Full - length sequence
XLOX2R-Hind3- F
GCTGCCTCTCTAAATCTGC
XLOX2R-Kpn1-R
TGACTCAATGAAACAGAC
