# Supplementary material for: Origin of Secretin Receptor Precedes the Advent of Tetrapoda: Evidence on the Separated Origins of Secretin and Orexin
Source: PLoS One. 2011 Apr 29;6(4):e19384. doi: 10.1371/journal.pone.0019384 (PMC3084839; doi:10.1371/journal.pone.0019384)
Supplement: Table S2 — Accession numbers of amino acid sequences of secretin/glucagon superfamily hormones and GPCR secretin family receptors [58], [59]. (PPTX) [file pone.0019384.s012.pptx]

## Slide 1
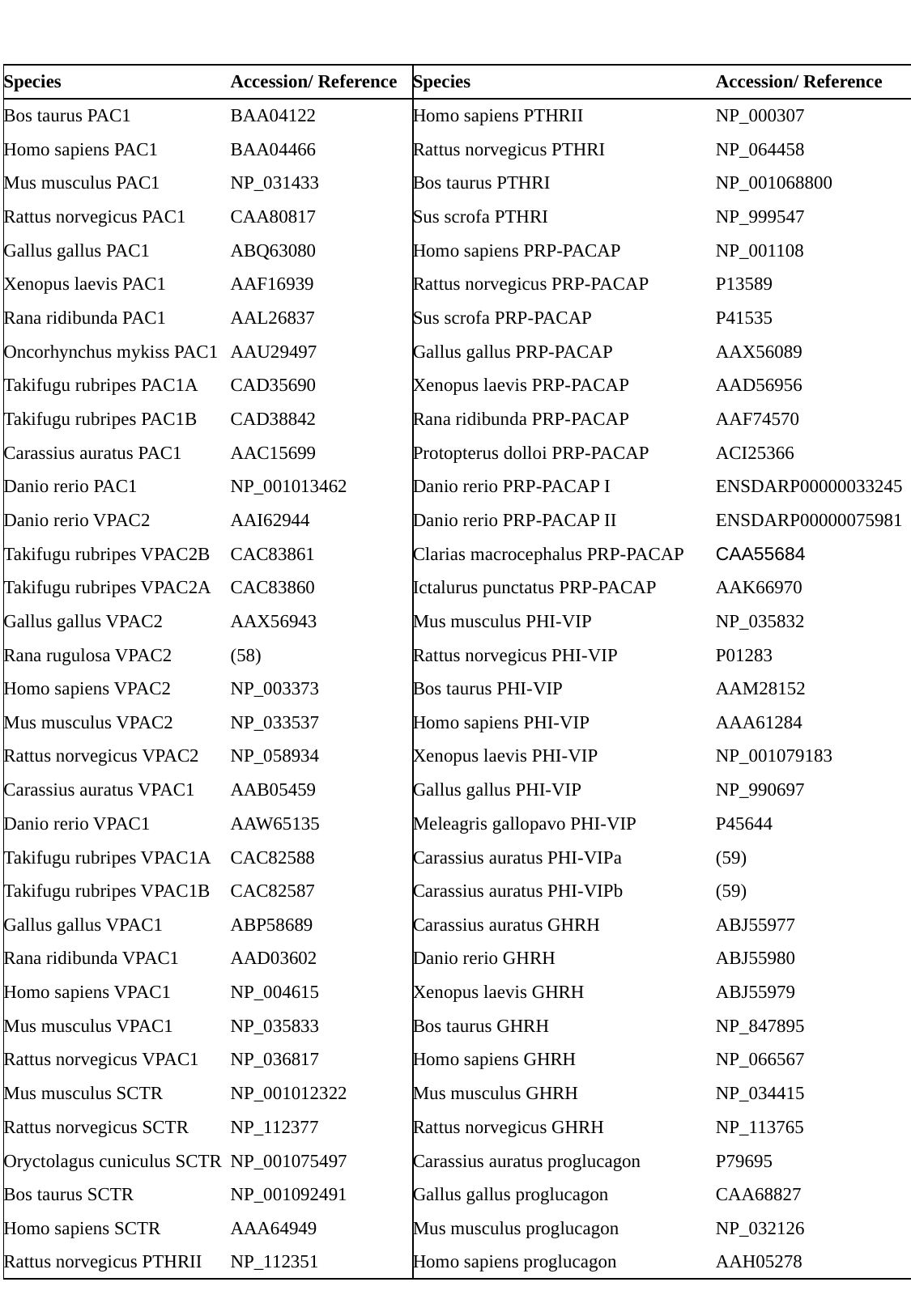

| Species | Accession/ Reference | Species | Accession/ Reference |
| --- | --- | --- | --- |
| Bos taurus PAC1 | BAA04122 | Homo sapiens PTHRII | NP\_000307 |
| Homo sapiens PAC1 | BAA04466 | Rattus norvegicus PTHRI | NP\_064458 |
| Mus musculus PAC1 | NP\_031433 | Bos taurus PTHRI | NP\_001068800 |
| Rattus norvegicus PAC1 | CAA80817 | Sus scrofa PTHRI | NP\_999547 |
| Gallus gallus PAC1 | ABQ63080 | Homo sapiens PRP-PACAP | NP\_001108 |
| Xenopus laevis PAC1 | AAF16939 | Rattus norvegicus PRP-PACAP | P13589 |
| Rana ridibunda PAC1 | AAL26837 | Sus scrofa PRP-PACAP | P41535 |
| Oncorhynchus mykiss PAC1 | AAU29497 | Gallus gallus PRP-PACAP | AAX56089 |
| Takifugu rubripes PAC1A | CAD35690 | Xenopus laevis PRP-PACAP | AAD56956 |
| Takifugu rubripes PAC1B | CAD38842 | Rana ridibunda PRP-PACAP | AAF74570 |
| Carassius auratus PAC1 | AAC15699 | Protopterus dolloi PRP-PACAP | ACI25366 |
| Danio rerio PAC1 | NP\_001013462 | Danio rerio PRP-PACAP I | ENSDARP00000033245 |
| Danio rerio VPAC2 | AAI62944 | Danio rerio PRP-PACAP II | ENSDARP00000075981 |
| Takifugu rubripes VPAC2B | CAC83861 | Clarias macrocephalus PRP-PACAP | CAA55684 |
| Takifugu rubripes VPAC2A | CAC83860 | Ictalurus punctatus PRP-PACAP | AAK66970 |
| Gallus gallus VPAC2 | AAX56943 | Mus musculus PHI-VIP | NP\_035832 |
| Rana rugulosa VPAC2 | (58) | Rattus norvegicus PHI-VIP | P01283 |
| Homo sapiens VPAC2 | NP\_003373 | Bos taurus PHI-VIP | AAM28152 |
| Mus musculus VPAC2 | NP\_033537 | Homo sapiens PHI-VIP | AAA61284 |
| Rattus norvegicus VPAC2 | NP\_058934 | Xenopus laevis PHI-VIP | NP\_001079183 |
| Carassius auratus VPAC1 | AAB05459 | Gallus gallus PHI-VIP | NP\_990697 |
| Danio rerio VPAC1 | AAW65135 | Meleagris gallopavo PHI-VIP | P45644 |
| Takifugu rubripes VPAC1A | CAC82588 | Carassius auratus PHI-VIPa | (59) |
| Takifugu rubripes VPAC1B | CAC82587 | Carassius auratus PHI-VIPb | (59) |
| Gallus gallus VPAC1 | ABP58689 | Carassius auratus GHRH | ABJ55977 |
| Rana ridibunda VPAC1 | AAD03602 | Danio rerio GHRH | ABJ55980 |
| Homo sapiens VPAC1 | NP\_004615 | Xenopus laevis GHRH | ABJ55979 |
| Mus musculus VPAC1 | NP\_035833 | Bos taurus GHRH | NP\_847895 |
| Rattus norvegicus VPAC1 | NP\_036817 | Homo sapiens GHRH | NP\_066567 |
| Mus musculus SCTR | NP\_001012322 | Mus musculus GHRH | NP\_034415 |
| Rattus norvegicus SCTR | NP\_112377 | Rattus norvegicus GHRH | NP\_113765 |
| Oryctolagus cuniculus SCTR | NP\_001075497 | Carassius auratus proglucagon | P79695 |
| Bos taurus SCTR | NP\_001092491 | Gallus gallus proglucagon | CAA68827 |
| Homo sapiens SCTR | AAA64949 | Mus musculus proglucagon | NP\_032126 |
| Rattus norvegicus PTHRII | NP\_112351 | Homo sapiens proglucagon | AAH05278 |
